# A Two-Stage Optimization Approach for Healthcare Facility Location- Allocation Problems With Service Delivering Based on Genetic Algorithm

**DOI:** 10.3389/ijph.2023.1605015

**Published:** 2023-02-28

**Authors:** Azadeh Salami, Behrouz Afshar-Nadjafi, Maghsoud Amiri

**Affiliations:** ^1^ Department of Industrial Engineering, Qazvin Branch, Islamic Azad University, Qazvin, Iran; ^2^ Department of Industrial Management, Faculty of Management and Accounting, Allameh Tabataba’i University, Tehran, Iran

**Keywords:** healthcare, hierarchical facility location-allocation, health service network design, multi-period, hybrid genetic algorithm

## Abstract

**Objective:** This study assesses a multi-period capacitated maximal-covering location-allocation model for healthcare services, taking interservice referral as well as equity access into account.

**Methods:** A two-stage optimization strategy is used to formulate the model. In the first stage, facilities are located to maximize covered demand, and in the second stage, patients are allocated to capacitated facilities based on their radius of coverage over multiple time periods. The problem, which belongs to the NP-hard class of optimization problems, is solved using a linear mixed-integer programming (MILP) model.

**Results:** A numerical example is presented to evaluate the efficiency of the proposed model. In addition, to identify near-optimal solutions for large instances, a hybrid genetic-sequential quadratic programming approach (GA-SQP) is developed. To examine the performance and efficiency of the GA-SQP, we employed several randomly generated test instances of various sizes and compared them to those obtained using the exact method.

**Conclusion:** The proposed model has demonstrated an excellent ability in locating healthcare facilities and allocating health services while taking shortage and equity into account during each time period.

## Introduction

Locating and allocating healthcare facilities has drawn lots of attention today. Most countries are concerned about providing adequate services. Models of location-allocation discover and locate new facilities in various geographic zones and then assign demand nodes to the discovered facilities. Maláková K. evaluated that every patient has the right to get the best professional-level healthcare services. The accessibility of healthcare is influenced by the distance to the medical facilities that are designated in accordance with the medical specialties or the specific types of services (1). The best location depends on the facility’s capacity, optimal distance, population density, etc. (2). The aim of solving location-allocation problems is to find the best location or locations for one or more facilities that will produce the highest utility value based on one or more criteria (3, 4). Location-allocation is a complex problem for decision-makers to solve. They require a decision-making tool to locate the facility based on several factors in order to effectively supply demand points (5). Several comprehensive reviews (6, 7) of facility location-allocation problems have been developed in the literature. Khodaparasti et al. (8) developed a multi-objective location allocation model to enhance community-based health programs in Iran that reflects social welfare concerns such as equity and local accessibility. Ahmadi-Javid et al. (9) presented a comprehensive review of healthcare facility locations in the last decade. They classify different types of non-emergency and emergency healthcare facility locations in terms of location management. Narula (10) has published reviews on the use of hierarchical location models. The proposed model incorporates this type of modeling. One of the first research works in the field of locating healthcare facilities was presented by Calvo and Marks (11). They used a total local hierarchy locating model, which since then has been tackled many times in the literature. Dökmeci (12) presented the first continuous approach model to locate hierarchical facilities for locating four different levels of healthcare facilities in one area. Narula and Ogbu (13) offered many heuristic approaches to handle a two-level hierarchical location-allocation problem with the potential for referral services. Malczewski and Ogryczak (14) developed a model for a multi-objective locating problem in healthcare facilities that is similar to the fuzzy optimization method. Marianov and Serra (15) sought to locate hierarchical facilities with random demands and congestion. Galvao et al. (16) proposed a total hierarchical system for locating service facilities for women and infants that considered only three facility levels. Mitropoulos et al. (17) developed a method based on a bi-objective mathematical programming model for locating hospitals and primary healthcare centers to improve their operational shortcomings. Şahin and Süral (18) provided a comprehensive review article and classified hierarchical locating into three categories. Detailed descriptions of hierarchical location-allocation problems are presented in (18, 19). Hodgson and Jacobsen (20) pointed out that locating total hierarchical facilities simultaneously had better results than locating these facilities individually. Mestre, Oliveira, and Barbosa-Póvoa (21) proposed a hierarchical model to maximize geographical access to a healthcare network by investigating the location and supply of center services. Shishebori and Babadi (22) developed an integrated mathematical model for locating and designing a network to improve the efficiency of healthcare services. Ghadiri and Jebelameli (23) investigated an uncapacitated facility location and network design in a multi-period state by considering budget constraints. To maximize perinatal treatment approachability, Baray and Cliquet (24) developed a hierarchical location-allocation model for maternity hospitals in France. Mohammadi et al. (25) investigated locating facilities and network design for a health network. Mestre et al. (26) formulated hierarchical location-allocation models that consider the uncertainty related to the demand and supply of healthcare services to minimize costs. Hasanzadeh and Bashiri (27) examined the establishment of relief centers to reduce relief operations and establish higher-level centers to minimize transfer time and costs. Shavarani (28) proposes a multi-level facility location-allocation problem to concurrently account for recharge stations, relief centers, and the number of required drones to cover all the demand for relief in a post-disaster period (29) investigated primary healthcare facility allocation in Finland. They applied p-median type location-allocation analysis to geographic information systems (GIS). Wang (30) proposed a two-stage optimization model for facility location-allocation problems of multiple facilities with continuous demand along the line. Ghasemi (31) studied blood supply chain location-allocation problems after a disaster and proposed a model for simultaneous midterm and short-term planning to minimize the network’s cost. Jenkins et al. (32) developed an integer mathematical programming formulation to determine the location and allocation of medical evacuation assets over the phases of a military deployment.

The location of the facilities preparing for public health service is very important and critical in ensuring that the chosen location network serves the purpose of minimizing social cost or equivalently maximizing the benefits to people. Cho (33) investigated the location and capacity of healthcare facilities, while Chu and Chu (34) determined the location of new hospitals in relation to existing hospitals. Cardoso et al. (35) investigated a time horizon and planned it periodically. Their primary purpose was to promote social equity in various ways. They expanded their model further to address social equity, taking into account uncertainty in the amount of demand as well as the length of service received (36). The quandary in healthcare is how to account for equity in service distribution. Equity may have an impact on the number of people covered and the services provided (37). Zhang et al. (38) examine the public healthcare location-allocation problem in Hong Kong and where such healthcare facilities should be located to improve the equity of accessibility, raise the total accessibility for the entire population, reduce the population that falls outside the coverage range, and decrease the cost of building new facilities. Essar, M.Y., et al. (39) concluded that in the pandemic situation, what is known as “health equity” may be nothing more than an illusion due to the unequal provision of health services. In this study, we consider equity in healthcare facility locations and service assignment based on the minimum number of higher-level healthcare centers (HLHCCs) and lower-level healthcare centers (LLHCCs) located at each demand point. We also consider an index that determines the difference between the highest and lowest percentage of shortages for each service in each period to distribute all services equally among all nodes.

The facility’s capacity influences the services it can provide. Therefore, the facility should be well located so that its capacity can accommodate all demands (40, 41). Cardoso et al. (35), based on the research conducted by Mestre et al. (21), presented a model for locating and determining service capacity in the treatment network. Vidyarthi and Kuzgunkaya (42) studied the location of preventive facilities concerning random demand and congestion at centers. Zhang and his colleagues (43) explored the possibility of locating the preventive facilities by considering the congestion issue. Pehlivan et al. (44) presented a model to determine the location and capacity of services for women and infants. Zhang et al. (45) examined the impact of patients’ preferences on the location and capacity of the centers.

Several metaheuristic solutions exist in the location-allocation arena. The Genetic Algorithm (GA), a materialistic search technique that uses the analogy of natural evolution in the search algorithm, is used in this study. It can find optimal or near-optimal solutions while staying out of the local optimals (46). Hosage and Goodchild (47) first identified the enormous potential of the genetic algorithm over heuristics in solving an uncertain class of location-allocation problems. Zhao et al. (48) proposed a multi-objective, hierarchical mathematical model, allied with an interleaved modified particle swarm optimization algorithm and a genetic algorithm. Kaveh et al. (49) explored a multiple-criteria decision-making approach for hospital location-allocation.

In this paper, a comprehensive two-stage multi-period maximum-covering location-allocation model for healthcare services with consideration of inter-service referrals, shortages, and equity is developed. According to the related literature, single-stage location-allocation models have received the most attention in the context of health service networks, while two-stage location-allocation models have received less attention. This paper studies the issue of two-stage location-allocation in healthcare services with capacity constraints. In the first stage, the model seeks to efficiently determine the locations of lower-level healthcare centers (LLHCCs) and higher-level healthcare centers (HLHCCs) and the levels of capacity of each service provided at these locations, which are strategic (long-term) decisions. In the second stage, allocation and inter-service referrals are executed for each time period, which are operational decisions. Strategic decisions are those that will have an impact for years or even decades after the project is completed. Once a strategic decision is made, it is unlikely to be changed in the near future and will necessitate significant investment. Operational decisions, which typically have a 1-year or even 1-day impact, are those that are adjusted more frequently in response to current external and internal conditions. In this model, these two strategic and operational decisions are divided into two stages, which make it easy to change the allocation and inter-service referrals in response to demand. Furthermore, no research in the literature has applied various relevant aspects of a health service network configuration, such as interservice referral, equity, capacitated constraints, and shortage, and their work was not a multiperiod investigation. Indeed, this study proposes a novel efficient mathematical model and a solution method to provide an integrated model for the two-stage capacitated location-allocation of healthcare services for the design of healthcare networks in relation to the aforementioned issues. Determining the capacity of a healthcare facility is usually a critical consideration. Pehlivan et al. (50) developed a novel hierarchical service network for determining the location and capacity of perinatal facilities. They defined a new hierarchical service network that excluded service referrals between centers. As a result, the major contribution of our study is the consideration of such inter-service referrals and bringing all these constraints to an exact optimization approach. It’s also worth noting that the developed model with these constraints is classified as an NP-hard problem. Therefore, using the exact method for large instances to find the optimal solution cannot be obtained in a reasonable time. To solve the model in a reasonable amount of time, we extend a hybrid GA, as recommended in Zarrinpoor et al (51). The contributions of this study are summarized as follows:

1. Developing mathematical models for the two-stage capacitated healthcare location-allocation problem, which includes two levels of facility and multi-services, as well as multi-capacity, shortage.
2. Considering service referral to ensure that demand receives the best possible healthcare close to home, as well as improving resource availability and quality of care at lower levels.
3. Considering equity in healthcare facility locations and service assignment based on the minimum number of HLHCCs and LLHCCs located at each demand point, in addition to distribute all services equally among all nodes, we consider an index that determines the difference between the highest and lowest percentage of shortages for each service in each period.
4. Developing an effective hybrid GA-SQP approach to solve the problem and evaluating its performance using numerical instances.

## Methods

**FIGURE 1:**
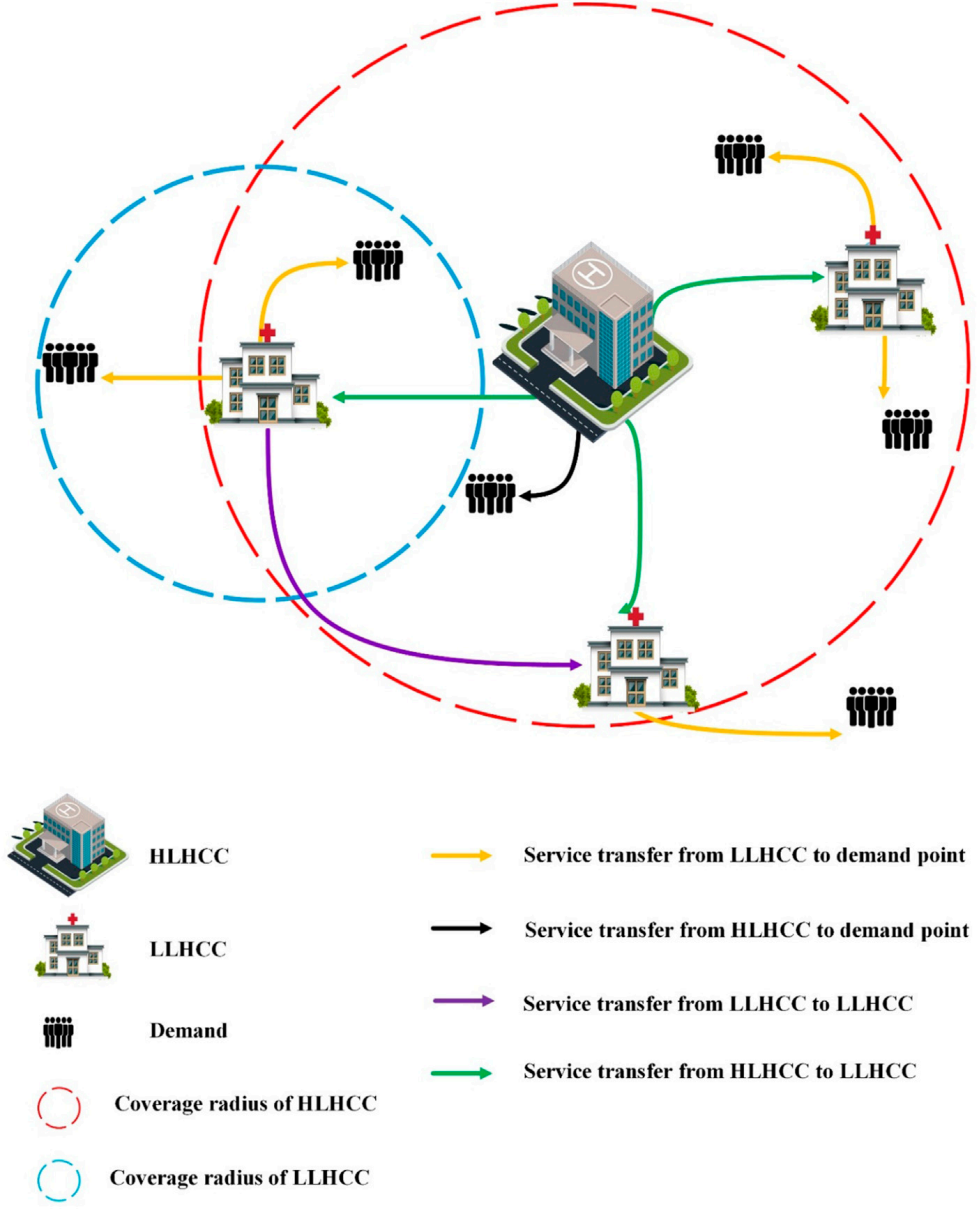
The schematic representation of the relevant hierarchical health service network (Iran, 2022).

A network is used to represent the model under investigation. Nodes show either candidate locations for LLHCCs or HLHCCs and demand concentrations, or both, because population centers are feasible locations for both health centers and patients. As seen in Figure 1, a two-level multi-flow nested hierarchy with service referral is considered. Our proposed model attempted to reduce patient transfer and service costs by establishing HLHCCs and LLHCCs based on demand points. In this case, each LLHCC should be placed within an HLHCC’s service coverage radius, and demand points should be within at least one LLHCC’s or HLHCC’s coverage radius. LLHCCs are also linked to improving demand responsiveness and equity. If the demand points are within the coverage radius of the LLHCC or HLHCC, the services are transferred.

A two-stage optimization technique is used to develop the proposed model. Two levels of facilities with different service categories and hierarchical properties are considered. The first stage involves the location of HLHCCs and LLHCCs, and the second stage involves allocating capacitated services over multiple time periods. A potential set of services for outpatient services has been considered at two levels. The first level is the HLHCC, which offers inpatient and emergency health services in addition to outpatient services and the second level is the LLHCC, which provides outpatient services. In other words, patients seeking outpatient services can go directly to the LLHCC or HLHCC outpatient department. The difference between the services available in the outpatient departments of the HLHCC and the LLHCC is the expertise of the physicians, facilities, and equipment. On the other hand, because of the importance of the bed in the hospitalization sector, the equipped bed is regarded as a service, and capacity is assigned to it. Given the nature of the inpatient ward, which refuses to accept patients when all beds are already occupied, the percentage of rejected patients has been considered a measure of social equity to minimize unmet demands for providing access to services to all people. Our model has considered the issue of a shortage of services, too. Also, we embarked on a plan to prioritize services so that the cost of a deficit should be regarded as higher for essential and emergency services. In other words, the model has minimized the shortage to achieve more social equity. In this network, patients can refer to any of the outpatient services available at the HLHCC or LLHCC if they are within their predetermined coverage radius. Also, due to insufficient expertise at the LLHCC, a percentage of patients are referred to the appropriate services at the HLHCC. However, patients with emergencies can only apply to the HLHCC emergency department. The patient’s hospitalization is only possible by referral from the outpatient and emergency departments of the HLHCC. The HLHCCs also provide some of the more specialized services that are not delivered by the LLHCCs. Health and treatment centers are among the most important facilities that directly contribute to individual and societal health. Every community needs easy, affordable, and timely access to these facilities. Thus, this study has considered the issue of transferring services among LLHCCs to provide better services. In this regard, LLHCCs that do not offer a specific service or have run out of it can receive that service from other LLHCCs within their coverage radius to provide better services. This procedure is important for two reasons. On the one hand, the patient can receive all the healthcare he or she needs from an LLHCC or HLHCC. On the other hand, LLHCCs and HLHCCs can provide all health services, which in turn reduces costs and meets all requirements better.

### Model Formulation

In this section, we define the assumptions, sets, parameters, and decision variables for our model, which is shown in Supplementary Material S1, and then provide the study mathematical formulas.

### Mathematical Model

The mathematical model is presented as follows, based on the defined assumptions and definitions:

### First Stage Formulation

$$\mathrm{Min}\overset{I}{\underset{k=1}{\sum }}F_{k}X_{kk}+\overset{I}{\underset{j=1}{\sum }}f_{j}y_{j}+\overset{I}{\underset{k=1}{\sum }}\overset{N}{\underset{n=1}{\sum }}\overset{M\left(n\right)}{\underset{m=1}{\sum }}G_{knm}z_{knm}$$ (1)

$$X_{jk}\leq X_{kk},\forall j,k$$ (2)

$$\overset{I}{\underset{\begin{array}{l} k=1\\ k\neq j \end{array}}{\sum }}X_{jk}=y_{j},\forall j$$ (3)

$$\overset{m\left(n\right)}{\underset{m=1}{\sum }}z_{knm}=X_{kk},\forall k,n$$ (4)

$$X_{ii}+y_{i}\leq 1,\forall i$$ (5)

$$X_{jk}\leq \overset{N}{\underset{n=1}{\sum }}b_{jkn}X_{kk}M,\forall j,k$$ (6)

$$\overset{I}{\underset{k=1}{\sum }}b_{ikn}X_{kk}\geq H,\forall i,n$$ (7)

$$\overset{I}{\underset{j=1}{\sum }}a_{ijn}y_{j}\geq V,\forall i,n$$ (8)

### Second Stage Formulation

$$\begin{array}{l} \mathrm{Min}\overset{I}{\underset{j=1}{\sum }}\overset{I}{\underset{j^{\prime }=1}{\sum }}\overset{T}{\underset{t=1}{\sum }}C_{jj^{\prime }}x_{jj^{\prime }t}+\overset{I}{\underset{j=1}{\sum }}\overset{I}{\underset{j=1}{\sum }}\overset{N}{\underset{n=1}{\sum }}\overset{T}{\underset{t=1}{\sum }}c_{jj^{\prime }n}^{\prime }\cdot h_{jj^{\prime }nt}+\overset{I}{\underset{i=1}{\sum }}\overset{I}{\underset{k=1}{\sum }}\overset{N}{\underset{n=1}{\sum }}\overset{T}{\underset{t=1}{\sum }}q_{kin}^{\prime }u_{kint}\\ +\overset{I}{\underset{j=1}{\sum }}\overset{I}{\underset{i=1}{\sum }}\overset{N}{\underset{n=1}{\sum }}\overset{T}{\underset{t=1}{\sum }}q_{jin}e_{jint}+\overset{I}{\underset{k=1}{\sum }}\overset{I}{\underset{i=1}{\sum }}\overset{N}{\underset{n=1}{\sum }}\overset{T}{\underset{t=1}{\sum }}c_{kjn}^{\prime \prime }w_{kjnt}+\overset{I}{\underset{i=1}{\sum }}\overset{N}{\underset{n=1}{\sum }}\overset{T}{\underset{t=1}{\sum }}P_{in}B_{\mathrm{int}} \end{array}$$ (9)

$$\overset{I}{\underset{j^{\prime }=1}{\sum }}\overset{T}{\underset{t=1}{\sum }}x_{jj^{\prime }t}\leq y_{j}M,\forall j$$ (10)

$$\overset{I}{\underset{j=1}{\sum }}\overset{T}{\underset{t=1}{\sum }}x_{jj^{\prime }t}\leq y_{j^{\prime }}M,\forall j^{\prime }$$ (11)

$$\overset{I}{\underset{j=1}{\sum }}w_{kjnt}+\overset{I}{\underset{i=1}{\sum }}u_{kint}\leq \overset{m\left(n\right)}{\underset{m=1}{\sum }}z_{knm}{\hat{c}}_{nm},\forall k,n,t$$ (12)

$$\overset{I}{\underset{n=1}{\sum }}h_{jj^{\prime }nt}\leq x_{jj^{\prime }t}M,\forall j,j^{\prime },t$$ (13)

$$\overset{N}{\underset{n=1}{\sum }}\overset{T}{\underset{t=1}{\sum }}w_{kjnt}\leq X_{jk}M,\forall j,k$$ (14)

$$\overset{I}{\underset{k=1}{\sum }}\overset{N}{\underset{n=1}{\sum }}\overset{T}{\underset{t=1}{\sum }}w_{kjnt}\leq y_{j}M,\forall j$$ (15)

$$\overset{I}{\underset{k=1}{\sum }}\overset{T}{\underset{t=1}{\sum }}\overset{I}{\underset{j=1}{\sum }}w_{kjnt}\leq \left(1-A_{n}\right)M,\forall n$$ (16)

$$\overset{T}{\underset{t=1}{\sum }}w_{kjnt}\leq b_{jkn}X_{kk}M,\forall j,k,n$$ (17)

$$\overset{T}{\underset{t=1}{\sum }}e_{jint}\leq a_{ijn}y_{j}M,\forall j,i,n$$ (18)

$$\overset{T}{\underset{t=1}{\sum }}u_{kint}\leq b_{ikn}X_{kk}M,\forall i,k,n$$ (19)

$$\overset{I}{\underset{k=1}{\sum }}w_{kjnt}+\overset{I}{\underset{j^{\prime }=1}{\sum }}h_{j^{\prime }jnt}\geq \overset{I}{\underset{i=1}{\sum }}e_{jint}+\overset{I}{\underset{j^{\prime }=1}{\sum }}h_{jj^{\prime }nt},\forall j,n,t$$ (20)

$$B_{\mathrm{int}}\geq D_{\mathrm{int}}-\overset{I}{\underset{j=1}{\sum }}e_{jint}-\overset{I}{\underset{k=1}{\sum }}u_{kint},\forall i,n,t$$ (21)

$$\alpha_{nt}=\max_{i}\left\{\frac{B_{\mathrm{int}}}{D_{\mathrm{int}}}\right\}-\min_{i}\left\{\frac{B_{\mathrm{int}}}{D_{\mathrm{int}}}\right\},\forall n,t$$ (22)

$$\alpha_{nt}\leq \alpha_{n}^{\max},\forall n,t$$ (23)

$$y_{j}\in \left\{0,1\right\},\forall j$$ (24)

$$X_{jk},X_{kk}\in \left\{0,1\right\},\forall j,k$$ (25)

$$x_{jj^{\prime }t}\in \left\{0,1\right\},\forall j,j^{\prime }$$ (26)

$$z_{knm}\in \left\{0,1\right\},\forall k,n,m$$ (27)

$$w_{kjnt},h_{jj^{\prime }nt},u_{kint},B_{\mathrm{int}},D_{\mathrm{int}},e_{jint},\alpha_{nt},\beta_{nt},\delta_{nt},\alpha_{n}^{\max}\geq 0,\forall k,i,j,j^{\prime },n,t$$ (28)

J,K,I,V,H,N,T≥0 (29)

Supplementary Material S2 contains descriptions of the first and second stages of mathematical modeling.

### Model Linearization

The presented mathematical model is non-linear because of constraint 22 in the second stage. Therefore, the new variables of $\beta_{nt}$ and $\gamma_{nt}$ are substituted with $\max_{i}\left\{\frac{B_{\mathrm{int}}}{D_{\mathrm{int}}}\right\}$ and $\min_{i}\left\{\frac{B_{\mathrm{int}}}{D_{\mathrm{int}}}\right\}$ , respectively, to linearize this constraint as follows:

$$\alpha_{nt}=\beta_{nt}-\gamma_{nt},\forall n,t$$ (30)

$$\beta_{nt}=\overset{I}{\underset{i=1}{\sum }}\beta_{\mathrm{int}}^{\prime }\left(\frac{B_{\mathrm{int}}}{D_{\mathrm{int}}}\right),\forall n,t$$ (31)

$$\overset{I}{\underset{i=1}{\sum }}\beta_{\mathrm{int}}^{\prime }=1,\forall n,t$$ (32)

$$\beta_{nt}\geq \frac{B_{\mathrm{int}}}{D_{\mathrm{int}}},\forall i,n,t$$ (33)

$$\gamma_{nt}=\overset{I}{\underset{i=1}{\sum }}\gamma_{\mathrm{int}}^{\prime }\left(\frac{B_{\mathrm{int}}}{D_{\mathrm{int}}}\right),\forall n,t$$ (34)

$$\overset{I}{\underset{i=1}{\sum }}\gamma_{\mathrm{int}}^{\prime }=1,\forall n,t$$ (35)

$$\gamma_{nt}\leq \frac{B_{\mathrm{int}}}{D_{\mathrm{int}}},\forall i,n,t$$ (36)

$$\beta_{\mathrm{int}}^{\prime },\gamma_{\mathrm{int}}^{\prime }\in \left\{0,1\right\},\forall i,n,t$$ (37)

Due to the above linearization, variables $\beta_{nt}$ and $\gamma_{nt}$ are written as a multiplication of two variables, which causes the model to become non-linear again. Thus, a new variable is presented as $\beta_{\mathrm{int}}^{\prime \prime }$ instead of the multiplication of two binary and continuous variables, $\beta_{\mathrm{int}}^{\prime }$ and $B_{\mathrm{int}}$ . On the other hand, a variable is considered as $\gamma_{\mathrm{int}}^{\prime \prime }$ for multiplying two variables, $\gamma_{\mathrm{int}}^{\prime }$ and $B_{\mathrm{int}}$ to make the constraints in linear form. By introducing the new variables, constraint (Eq. 22) should be replaced by (Eq. 38), and the following additional constraints should be added to the proposed model:

$$\alpha_{nt}=\beta_{nt}-\gamma_{nt},\forall n,t$$ (38)

$$\beta_{nt}=\overset{I}{\underset{i=1}{\sum }}\frac{\beta_{\mathrm{int}}^{\prime \prime }}{D_{\mathrm{int}}},\forall n,t$$ (39)

$$\beta_{\mathrm{int}}^{\prime \prime }\geq B_{\mathrm{int}}-\left(1-\beta_{\mathrm{int}}^{\prime }\right)M,\forall i,n,t$$ (40)

$$\beta_{\mathrm{int}}^{\prime \prime }\leq B_{\mathrm{int}}+\left(1-\beta_{\mathrm{int}}^{\prime }\right)M,\forall i,n,t$$ (41)

$$\beta_{\mathrm{int}}^{\prime \prime }\leq \beta_{\mathrm{int}}^{\prime }M,\forall i,n,t$$ (42)

$$\overset{I}{\underset{i=1}{\sum }}\beta_{\mathrm{int}}^{\prime }=1,\forall n,t$$ (43)

$$\beta_{nt}\geq \frac{B_{\mathrm{int}}}{D_{\mathrm{int}}},\forall i,n,t$$ (44)

$$\gamma_{nt}=\overset{I}{\underset{i=1}{\sum }}\frac{\gamma_{\mathrm{int}}^{\prime \prime }}{D_{\mathrm{int}}},\forall n,t$$ (45)

$$\gamma_{\mathrm{int}}^{\prime \prime }\geq B_{\mathrm{int}}+\left(1-\gamma_{\mathrm{int}}^{\prime }\right)M,\forall i,n,t$$ (46)

$$\gamma_{\mathrm{int}}^{\prime \prime }\leq B_{\mathrm{int}}-\left(1-\gamma_{\mathrm{int}}^{\prime }\right)M,\forall i,n,t$$ (47)

$$\gamma_{\mathrm{int}}^{\prime \prime }\leq \gamma_{int}^{\prime }\;M,\forall i,n,t$$ (48)

$$\overset{I}{\underset{i=1}{\sum }}\gamma_{int}^{\prime }=1,\forall n,t$$ (49)

$$\gamma_{nt}\leq \frac{B_{\mathrm{int}}}{D_{\mathrm{int}}},\forall i,n,t$$ (50)

$$\beta_{int}^{\prime },\gamma_{int}^{\prime }\in \left\{0,1\right\},\forall i,n,t$$ (51)

## Results

A simple numerical example is used to demonstrate the applicability of the proposed model, as illustrated in Supplementary Material S3.

### Solution Methodology

**FIGURE 2:**
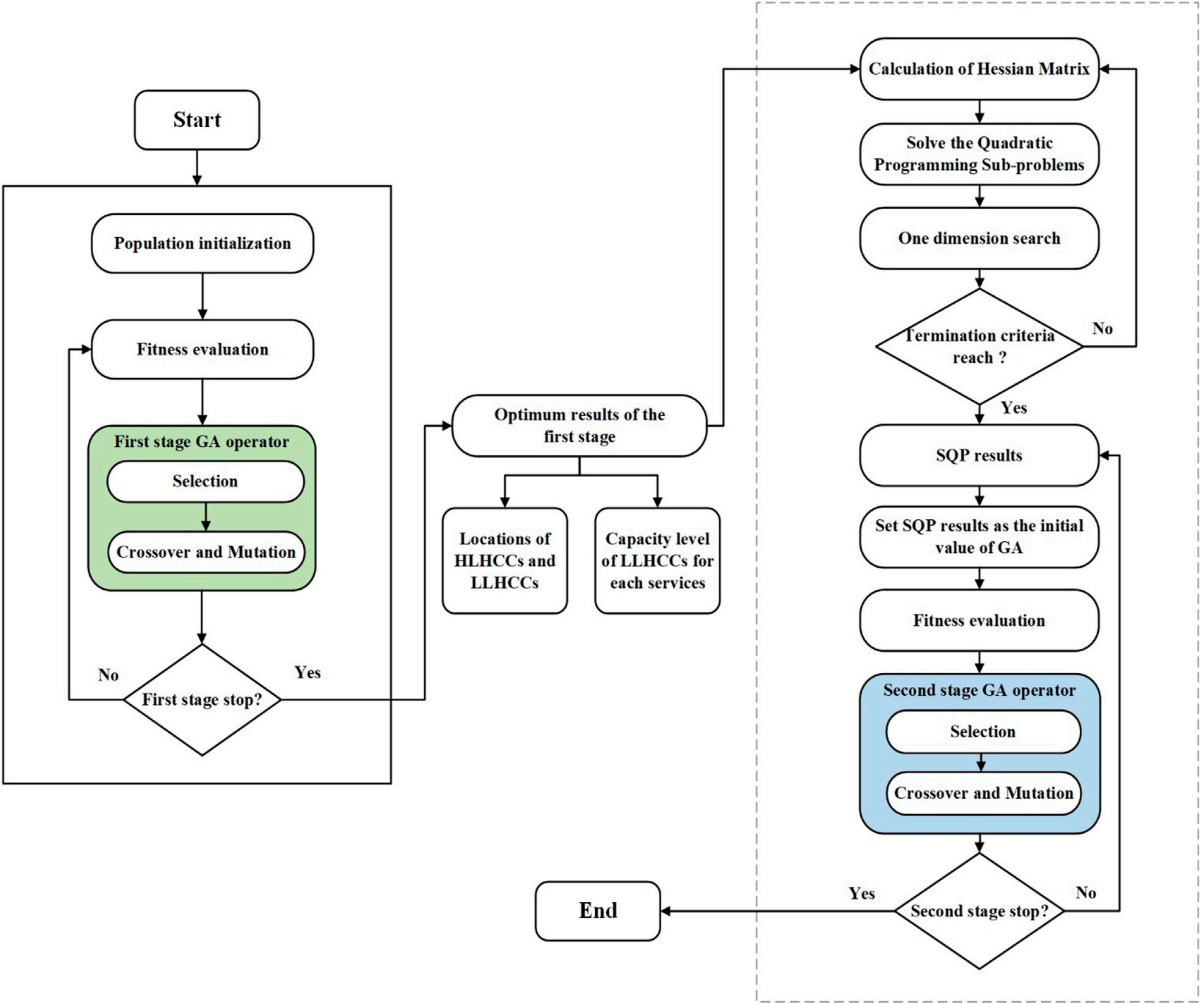
Two-stage Genetic-Sequential quadratic programming flowchart (Iran, 2022).

The location allocation problem is an NP-hard problem that can theoretically be solved using the branch and bound (linear programming) method. However, branch and bound is impractical due to the non-linearity and large scale of these problems. These factors have favored heuristic and metaheuristic solutions to the problem of location allocation (52). Exact methods for large instances are computationally expensive. Conventional methods are unable to solve the optimization problem within a reasonable time. As a result, several researchers used a variety of meta-heuristic algorithms to achieve near-optimal solutions. This type of algorithm is typically used to solve problems for which no suitable problem-specific algorithm or heuristic exists (53). A two-stage hybrid algorithm known as GA-SQP is used in the proposed model, which combines a genetic algorithm (GA) with sequential quadratic programming (SQP). GA is the main optimizer, while SQP significantly increases the power of the GA in terms of solution quality and speed of convergence to the optimal solution in the second stage. GA is a probabilistic, bio-inspired search method based on the natural selection approach (46). Gen et al. (54) demonstrated that GA would be the best approach for obtaining near global solutions. The following sections explain how we apply the GA-based algorithm to each stage of the proposed model. Figure 2 depicts the proposed GA-SQP algorithm’s flow chart.

### First Stage Initialization

**FIGURE 3:**
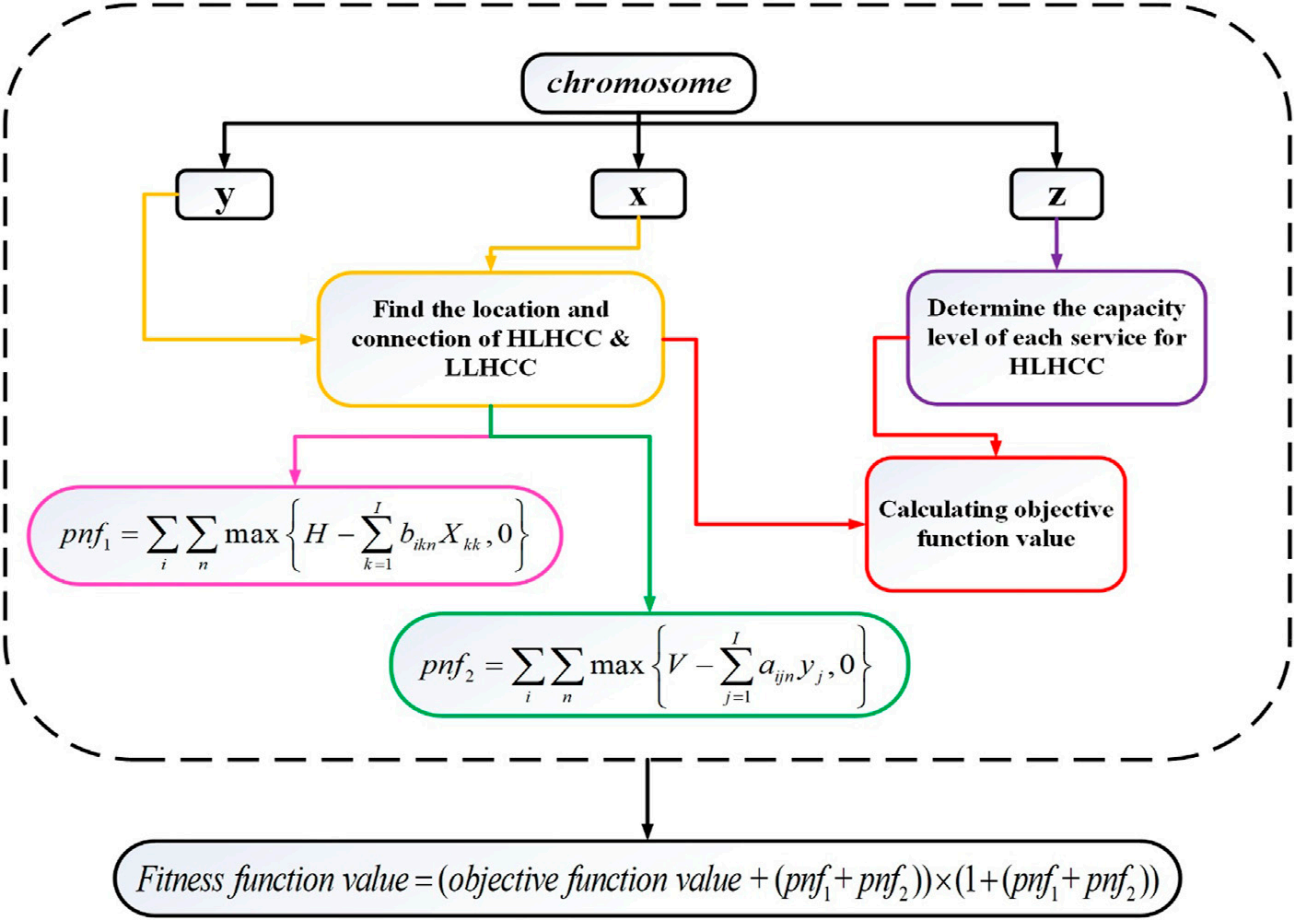
First stage decoding process flowchart (Iran, 2022).

In the first stage, GA searches for the near global optimum in the whole solution region to find the optimal locations of HLHCCs and LLHCCs. Furthermore, the capacity level of each service provided by LLHCCs and the connection of LLHCCs to HLHCCs are also determined. The first stage of the solution representation’s coding procedure involves encoding and decoding, which is shown in Supplementary Material S4. The decoding process is schematically illustrated in Figure 3, which includes valuing policies for dependent variables as well as the penalty function (pnf) mechanism for constraint handling.

#### Selection Operator

The selection process discovers chromosomes with higher fitness functions, which might generate better offspring to form the next generation. In this study, the selection process is based on the roulette wheel method (55) because of its efficiency and ease of implementation (56).

#### Crossover Operator

The crossover operator is vital in generating new generations. This operator locally searches for better offspring. Percentage chance of crossover (Pc) is a predetermined parameter based on problem size. The chromosome structure influences the choice of crossover operator. This study used a continuous type of uniform crossover (54). To ascertain this operator, a Mask matrix of the same size as one of the selected parent chromosomes is defined. This matrix’s entries are uniform numbers between 0 and 1 that are randomly generated. A convex combination of the corresponding gene in the created matrix and the associated genes in two selected parents should be used to calculate each gene (57). In other words, the genes of the two children with the same matrix size as one of their parents are generated using $Parent_{1}\times Mask+Parent_{2}\times \left(1-Mask\right)$ and $Parent_{1}\times \left(1-Mask\right)+Parent_{2}\times Mask$ .

#### Mutation Operator

After the crossover, a mutation occurs. This operator enhances a percentage of the genes on specific chromosomes and prevents algorithms from getting trapped in the locality. This study used a random uniform mutation, which is based on a Gaussian distribution. This operator generates a matrix of random numbers that are uniformly distributed between zero and one for each chromosome segment. Genes with mutation rates below a certain threshold are candidates for mutation operations, which alter the gene’s value.

### Second Stage Initialization

**FIGURE 4:**
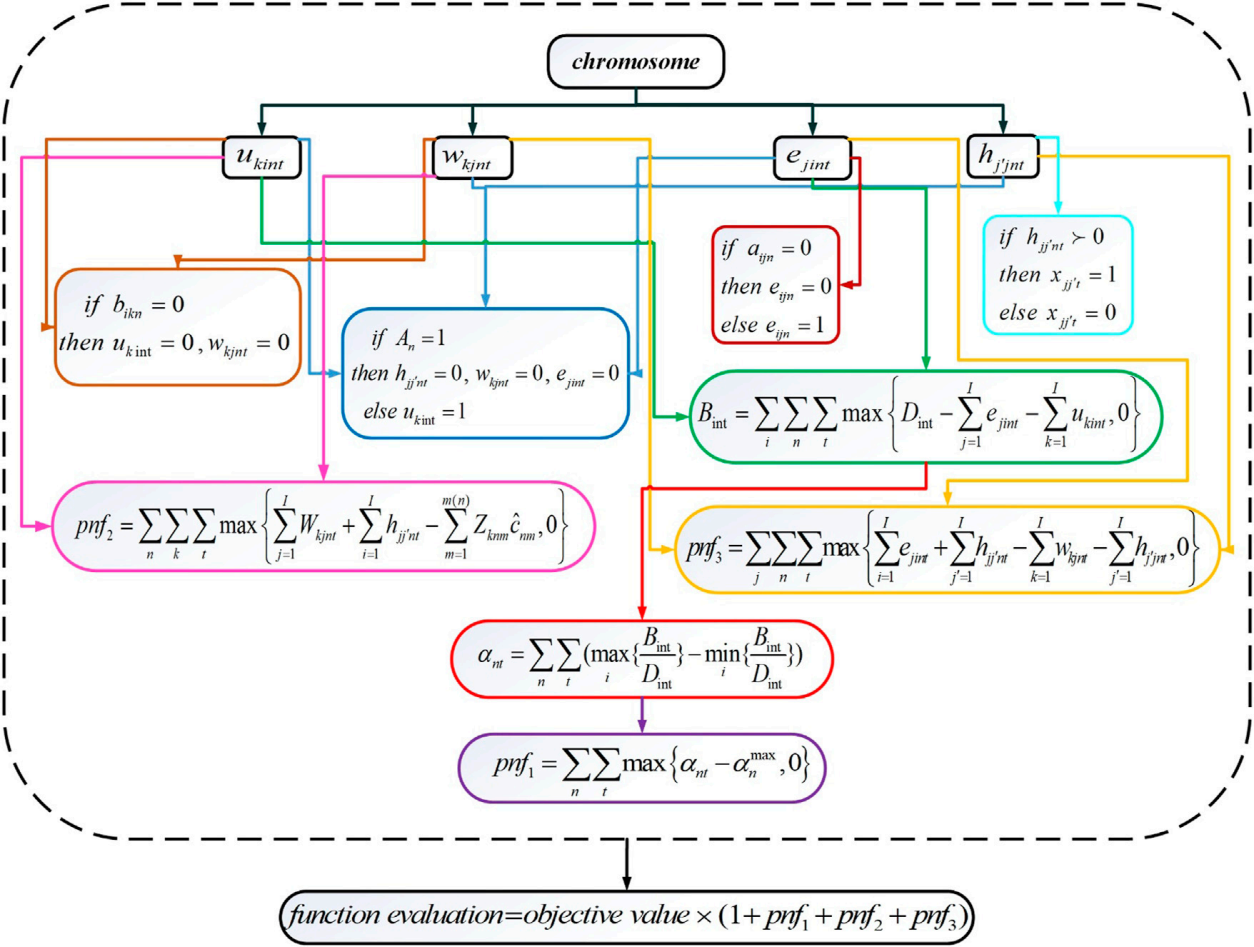
Second-stage decoding process flowchart (Iran, 2022).

HLHCCs and LLHCCs have been located in the first stage. In the second stage, the allocation of services and service transfers while considering the shortage are examined. Further, the achievement of equity access is ensured. When it comes to tackling non-linearly constrained optimization problems, SQP has undoubtedly proven to be the most successful method available. SQP is not a single algorithm, as is the case with most optimization approaches, but rather a conceptual method from which a large number of individual algorithms have been developed (58). The proposed GA-SQP hybrid method eliminates the need to provide a suitable starting point and allows for the assurance of a faster convergence speed and higher convergence accuracy to discover the optimal solution. First, SQP searches for the optimum in the whole solution region to provide a suitable starting point. Then the near-global optimal solution can be obtained by GA. The mathematical model developed in this study is presented as a two-stage optimization that separates strategic and operational decisions, the global optimal solution for the presented two-stage problem cannot be obtained, but the global optimal solution for each of the stages can be obtained. This is because a mixed-integer linear model is present at each stage. The encoding and decoding of the second stage are attached in Supplementary Material S4. Figure 4 schematically depicts the decoding process.

#### Selection Operator

The selection process in the second stage, like the first, is based on the roulette wheel method. Also, the continuous type of uniform crossover and mutation that was discussed in the first stage is used.

#### Elitism Process

The chromosomes that do not undergo crossover or mutation are given the elitism process. Elitism is being used to ensure that the quality of the solutions generated by the GA will not deteriorate with each new generation.

#### Stopping Criterion

After a predefined number of generations, the GA-SQP solution scheme is terminated. The size of the problem determines the rational number.

#### Parameters Tuning

The values of any meta-heuristic algorithm’s parameters determine its effectiveness and quality. Different parameter combinations in an algorithm can produce solutions of varying quality. In this study, the Taguchi approach is used to tune the algorithmic parameters. Taguchi is an efficient method that was created as an alternative to full fractional experimental design (59). The tuning of the parameters is described in Supplementary Material S5.

## Discussion

This section analyzes the efficiency of the proposed algorithms through sensitivity analysis. In the proposed model, V and H are vital factors that influence the fitness value. These factors illustrate the minimum number of LLHCCs and HLHCCs that must be covered by all nodes for any kind of service, respectively. All the factors mentioned above satisfy the measure of equity. If the planners have easing finances, they would like to select a solution with higher equity, accessibility, and coverage, even if this solution costs more. In verse, in straining finances, the choice will be completely different. Therefore, planners or the government can select disparate solutions within a different situation. The sensitivity analysis is fully described in Supplementary Material S6.

### Conclusion and Future Research

The main purpose of paying close attention to the location-allocation of healthcare facilities is to enhance community health by providing access to and delivery of high-quality services that meet people’s health needs. This paper studies the issue of two-stage location-allocation in healthcare services with capacity constraints. The model aims to efficiently determine the locations of LLHCCs and HLHCCs, as well as the levels of capacity of each service provided at these locations, in the first stage. Allocations and inter-service referrals are executed for each time period in the second stage. We separated strategic decisions from operational decisions by using a two-stage model. Strategic decisions are made in the first stage, and these are the decisions we make when we are finding or updating the locations of HLHCCs and LLHCCs, as well as when we are establishing these centers. These are long-term and expensive management strategies. Operational decisions, on the other hand, occur in the second stage, which involves medium-term decisions that allow for easy changes in allocation and inter-service referrals in response to demand. Since the shortage is allowed in the model, social equity is implemented in this network to balance the various shortfalls in all treatment centers. The proposed MILP model is a non-linear one that is converted into a linear one, and finally, the upper and lower boundaries for the problem are calculated using the branch and bound approach. Due to the complexity of our proposed model, for large problems, the exact approach might not be able to find the optimal solution in a reasonable time. Therefore, the two-stage hybrid GA-SQP algorithm is proposed to achieve optimal solutions effectively and efficiently. The applicability and efficiency of our proposed solution scheme are demonstrated by a comparison of the established algorithm with the exact solutions (B&B) in small and medium-sized test instances. Furthermore, a sensitivity analysis was performed on the impact of the minimum number of LLHCCs and HLHCCs that must be covered by all nodes on the objective functions. Although the proposed hybrid GA-SQP algorithm has demonstrated an excellent ability to solve the problem in a reasonable amount of time, there is still much opportunity to develop other hybrid strategies to solve the same problem in order to evaluate the strengths of various approaches in solving a problem of this type (60–62). Furthermore, the proposed hybrid algorithm provides a variety of options and parameter settings that are worth fully examining.

Future research could improve by considering non-spatial aspects to determine facility locations. For instance, people in a poor socioeconomic area may be unable to cover their healthcare costs even though a new hospital has been allocated to the neighborhood. Thus, the model could be expanded by altering the objective function and adding constraint sets to discover more socio-economic aspects in the formulation. Another valuable avenue for future research is to consider disaster scenarios that can be applied to the proposed model. This will be a valuable addition to our research’s ability to model real-world problems. Another aspect of future work could concentrate on the conditions of uncertainty that can be modeled as fuzzy sets. For example, demands that are deterministic in this paper can be conducted under the conditions of demand uncertainty.
