# Supplementary material for: A Two-Stage Optimization Approach for Healthcare Facility Location- Allocation Problems With Service Delivering Based on Genetic Algorithm
Source: Int J Public Health. 2023 Feb 28;68:1605015. doi: 10.3389/ijph.2023.1605015 (PMC10011119; doi:10.3389/ijph.2023.1605015)
Supplement: Supplementary file 1 [file DataSheet2.pdf]

First Stage Formulation

The objective function (1) minimizes the fixed cost of locating LLHCC and HLHCC and the cost of providing type n service to HLHCC at a m capacity level. Constraint (2) ensures that service transfer from HLHCC k to LLHCC j occurs when HLHCC k has been established. Constraint (3) guarantees that if an LLHCC has been established at node j, that LLHCC must be connected to one and only one HLHCC. Constraint (4) defines the concept of HLHCC capacity so that, if k HLHCC is established at node k, then for each type of service, the capacity level should be provided. Constraint (5) ensures that each node cannot be both the LLHCC and the HLHCC at the same time. Constraint (6) indicates that the LLHCC j can receive the nth service from HLHCC k if an HLHCC has been established at node k and the LLHCC j is within the covering radius of HLHCC k. Constraints (7) and (8) consider the equity of receiving each type of service by specifying a minimum number of LLHCC and HLHCC that should be established within a covering radius of each node of demand, respectively.

Second Stage Formulation

The objective function in Equation (9) minimizes the cost of connecting LLHCCs, the cost of transferring services from one LLHCC to another, the cost of providing service from HLHCC to demand point i, the cost of providing service from LLHCC to demand point i, the cost of transfer of type n service from HLHCC to an LLHCC, and the cost of type n service shortage for demand point i in period t. Constraints (10) and (11) ensure that transfers from LLHCCs j to j' are permitted if and only if LLHCCs j and j' have already been established at nodes j and j' during period t. Constraint (12) indicates that the amount of a particular type of service an HLHCC provides to all LLHCCs and patients in period t must be less than the capacity level of that HLHCC. Constraint (13) specifies the transfer of the nth type of service from LLHCC j to LLHCC j' in period t. Such

transfer is possible if these LLHCCs are connected. Constraint (14) represents that the HLHCC can provide services to an LLHCC in period *t* if the LLHCC is connected to the HLHCC. Constraint (15) indicates that the HLHCC can provide services to an LLHCC if the LLHCC has been established in period *t*. Constraint (16) does not allow the transfer of specialized services provided only in HLHCCs to the LLHCC in period *t*. Constraint (17) makes explicit that the jth LLHCC can only receive the nth service from the kth HLHCC in period *t* when an HLHCC has already been set up at the node *k*. Also, the jth LLHCC lies within the covering radius of *k* HLHCC. Constraint (18) ensures that the transfer of the nth service from the LLHCC to the patient is possible only if the patient is within the covering radius of that LLHCC in period *t*. Constraint (19) indicates that the jth patient from HLHCC *k* can receive the nth service if an HLHCC has been established at node *k* and the jth patient is within the covering radius of HLHCC k in period *t*. Constraint (20) is a balance constraint in period *t* for LLHCCs that ensures that each LLHCC's input must be higher than its output. That is, the services that an LLHCC receives from HLHCCs and other LLHCCs must be greater than the services it provides to its patients and other LLHCCs. Constraint (21) is the constraint of satisfying the demand in period *t*, which indicates the possibility of shortages in the model. In constraint (22), an index for evaluating social equity, α_{nt} has been introduced. This index calculates the difference between the highest and lowest percentage of deficiencies for each service over a given time period (in fact, we strive to distribute all services across all nodes to ensure social equity). Constraint (23) ensures social equity by defining the maximum difference between the maximum and the minimum value of the nth service shortage in all nodes. Equations (24) through (28) enforce binary and non-negativity restrictions on the corresponding decision variables.
