# Supplementary material for: A Two-Stage Optimization Approach for Healthcare Facility Location- Allocation Problems With Service Delivering Based on Genetic Algorithm
Source: Int J Public Health. 2023 Feb 28;68:1605015. doi: 10.3389/ijph.2023.1605015 (PMC10011119; doi:10.3389/ijph.2023.1605015)
Supplement: Supplementary file 2 [file DataSheet4.pdf]

Encoding Scheme of the First Stage

The most important part of using metaheuristic methods as solution schemes in practical optimization models is to do proper constraint handling so that they can be satisfied appropriately. To handle the constraints and implement the coding procedures in the first stage, three matrices are defined. In other words, each possible solution (chromosome) is introduced by three matrices: (a) the location matrix (y); (b) the assigning LLHCC to HLHCC matrix (x); and (c) the HLHCC capacity level matrix (z). The procedures for representing these matrices are described as follows:

i. The initial part of a chromosome is a $1 \times I$ matrix. (I) is the set of demand nodes. Each element of this matrix has a discrete uniform value of 0, 1, or 2, where 0 represents demand nodes, 1 represents LLHCCs, and 2 represents HLHCCs, which are selected randomly.

ii. The second part of a chromosome is a $1 \times I$ matrix that represents the connection of each LLHCC to the HLHCC (depending on the HLHCC's existence in that node). Each entry is generated based on a random discrete uniform value between 0 and I.

iii. The final part of a chromosome is a $N \times I$ matrix (N indicates the set of services) that demonstrates the HLHCC capacity level of the provided services. These matrix entries are uniformly chosen from random discrete values between 1 and m (m indicates the service capacity level for HLHCCs).

The following example further investigates the solution representation thoroughly, in which the process of finding all locations of HLHCCs and LLHCCs and their connections is explained. The matrices x, y, and z and the schematic network of the feasible solution are shown in Figure 1. As it is illustrated in this figure, there are ten demand nodes (I = 10). According to matrix y, nodes 5 and 7 and nodes 3, 4, and 6 represent the locations of HLHCCs and LLHCCs, respectively. As LLHCC nodes must be assigned to only one HLHCC node, node 4 and node 6 are assigned to node

5 and node 7, respectively. Node 3 should also be assigned to an HLHCC, but since there is none available in node 8, an LLHCC will not be established in node 3. In this example, 3 services (n = 3) and 2 capacity levels (m = 2) are assumed. In part b, the capacity level for established HLHCCs (nodes 5 and 7) is provided.

a.

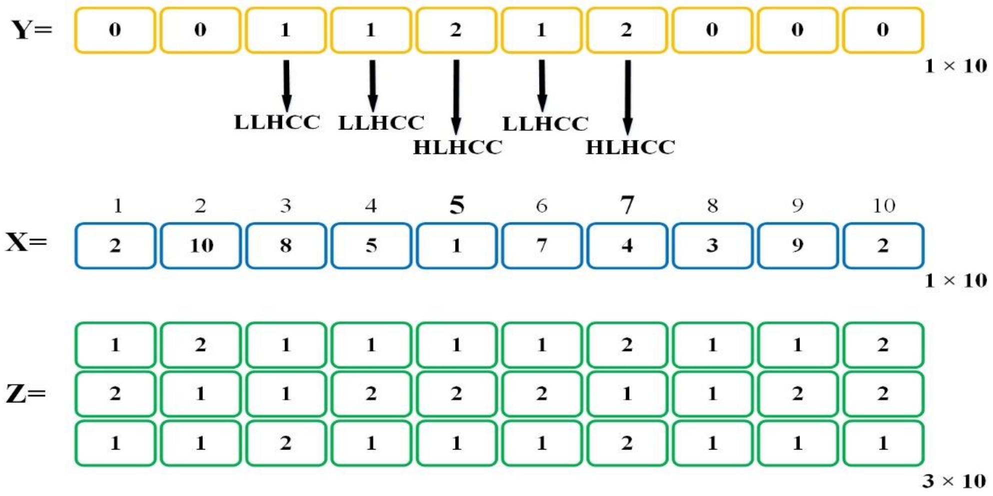

b.

	Service type		
Capacity level	1	2	3
5	1	2	1
7	2	1	2

c.

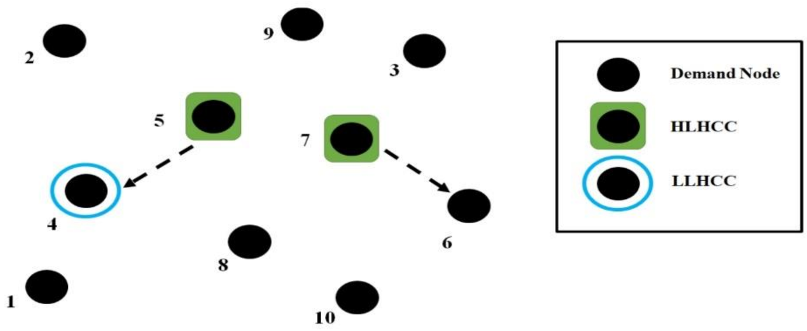

Figure 1 | The solution representation for the explained example (Iran, 2022)

Decoding Scheme of the First Stage

After generating the three matrices that have been described in the previous section, the objective function is computed by equation (1) in the first stage of our proposed model. All the constraints in the proposed chromosome structure are satisfied except constraints (7) and (8). Therefore, two penalty functions (pnf_1 and pnf_2) that correspond to satisfying these constraints are considered. pnf_1 and pnf_2 are the total violations of constraints related to the minimum number of HLHCC and LLHCC that should be established within the covering radius of each demand node, respectively. All this information can be used to find the fitness function value for the respective solution (chromosome).

Encoding Scheme of the Second Stage

In the second stage, the chromosome is associated with four key decision variables (u_{kint}, e_{jint}, W_{kjnt}, $h_{jj'nt}$). They are presented in four matrices as follows:

i. The genes associated with the amount of the nth type of service provided by the kth HLHCC to the ith demand point in period t (u_{kint}) are coded in the first part of the chromosome, using a $K \times I \times N \times T$ matrix. Each entry in this matrix contains a random number between 0 and D_{int}.

ii. The genes associated with the amount of nth type of service provided from the jth LLHCC to the ith demand point in period t (e_{jint}) are coded in the second part of the chromosome, using a $J \times I \times N \times T$ matrix. Each entry in this matrix contains a random number between 0 and $Max(D_{\text{int}} - \sum_{k=1}^{I} u_{kint})$.

iii. The genes associated with the amount of the nth type of service transfer from the kth HLHCC to the jth LLHCC in period t (W_{kjnt}) are coded in the third part of the chromosome using a

$K \times J \times N \times T$ matrix. Each entry in this matrix contains a random number between 0 and $Max(\hat{c}_{nm} - \sum_{i=1}^{I} u_{kint})$.

iv. The genes associated with the amount of the nth type of service transfer from the jth LLHCC to the j'^{th} LLHCC in period t ($h_{jj'nt}$) are coded in the last part of the chromosome using a $J \times J' \times N \times T$ matrix. Each entry in this matrix contains a random number between 0 and $Max(\sum_{k=1}^{I} w_{kjnt} - \sum_{i=1}^{I} e_{jint})$.

The proposed chromosome structure in the second stage satisfies all the constraints except constraints (12), (20), and (23). Besides, three modified policies are being considered. The way these constraints are dealt with is explained in the decoding section.

Decoding Scheme of the Second Stage

Based on the four matrices generated in the previous section, the allocation of services in each period can be determined while all constraints are considered. The objective function is computed by equation (9). Two penalty functions corresponding to soft constraints (15) and (16) are estimated based on the minimum number of HLHCCs and LLHCCs that must be covered by all nodes for each type of service. This data can be used to find the fitness function value for the respective solution (chromosome).

Since some generated chromosomes may not be feasible due to constraints in the proposed model's second stage, modifications and penalty functions are considered. For instance, in each period if $A_n = 1$ Then, all the operations belonging to the transfer of specialized services provided only in HLHCCs to the LLHCCs are deactivated (see Constraints 16). Furthermore, if $b_{ikn} = 0$, $a_{ijn} = 0$, constraints (17) and (18) require special attention to ensure that in each period, the allocation of

services is possible only if the patients and LLHCCs are within the covering radius of that center. The other variables are computed based on their corresponding constraints. In this regard, by specifying the value of $h_{jj'nt}$, the value of $x_{jj't}$ is obtained. Then, according to Constraints 21, B_{int} would be equal to the maximum value of $D_{\mathrm{int}} - \sum_{j=1}^{I} e_{jint} - \sum_{k=1}^{I} u_{kint}$. Based on B_{int}, the values of α_{nt} will be obtained in each period by determining the difference between the maximum and the minimum value of the nth service shortage in all nodes $(\alpha_{nt} = \max_i\{\frac{B_{\mathrm{int}}}{D_{\mathrm{int}}}\} - \min_i\{\frac{B_{\mathrm{int}}}{D_{\mathrm{int}}}\})$, constraints (22). In addition, pnf_1, pnf_2, and pnf_3 are specified, corresponding to constraints (23), (12), and (20), respectively. These values are defined as penalties by decreasing their fitness values in proportion to their degree of constraint violation for objective function evaluation.
