# Supplementary material for: A Two-Stage Optimization Approach for Healthcare Facility Location- Allocation Problems With Service Delivering Based on Genetic Algorithm
Source: Int J Public Health. 2023 Feb 28;68:1605015. doi: 10.3389/ijph.2023.1605015 (PMC10011119; doi:10.3389/ijph.2023.1605015)
Supplement: Supplementary file 3 [file DataSheet6.pdf]

In this section, the Lingo and GA-SQP results are compared with various H and V values. It should be noted that LINGO solves optimization models using the branch and bound (B&B) algorithm. Table 1 shows the values of the objective functions achieved using LINGO ($W_{B\&B}$), the solution scheme based on GA-SQP ($W_{GA\text{-}SQP}$), and the CPU time (in seconds) for all problems created. In addition, the last column shows the gap between $W_{B\&B}$ and $W_{GA\text{-}SQP}$, which is calculated using the following equation:

$$gap = \left(\frac{W_{GA-SQP} - W_{B\&B}}{W_{B\&B}}\right) \times 100 \tag{1}$$

Table 1 | Experimental results (Iran, 2022)

**Size (*I*, *N*, *M*, *T*)**	**Instance**	***H***	***V***	**Stage 1**				**Stage 2**				**Gap**
				**CPU Time**		**Objective value**		**CPU Time**		**Objective value**		
				**B&B**	**GA**	**B&B**	**GA**	**B&B**	**GA**	**B&B**	**GA**	
**(8, 2, 2, 2)**	A1	1	1	0.11	3.03	156	156	0.42	7.92	496	496	0.00
	A2	1	2	0.08	3.17	178	178	0.27	12.22	483	483	0.00
	A3	1	3	0.09	3.28	206	206	0.28	15.37	454	454	0.00
	A4	2	1	0.09	3.27	445	445	0.19	21.71	408	408	0.00
	A5	2	2	0.08	3.34	495	495	0.27	20.38	387	387	0.00
	A6	3	1	0.08	3.19	653	653	0.17	19.44	448	448	0.00
**(10, 3, 2 ,3)**	B1	1	1	0.11	4.37	157	157	0.94	21.34	2423	2423	0.00
	B2	1	2	0.16	4.21	171	171	0.66	54.66	2490	2520	0.00
	B3	1	3	0.12	4.28	184	184	0.89	62.73	2378	2439	2.56
	B4	1	4	0.11	4.47	210	210	1.55	80.54	2350	2417	2.85
	B5	2	1	0.12	4.31	372	372	0.7	63.44	1974	1974	0.00
	B6	2	2	0.14	4.29	384	384	0.56	88.61	1944	1944	0.00
	B7	2	3	0.14	4.42	410	410	0.98	93.84	1855	1855	0.00
	B8	3	1	0.52	4.21	670	670	1	82.48	1535	1535	0.00
	B9	3	2	0.19	4.14	692	692	0.5	112.27	1407	1452	3.19
	B10	4	1	0.14	4.57	892	892	0.53	120.49	1508	1570	4.11
	C1	1	1	166.82	14.05	864	864	176.85	4.32	25235	25235	0.00
	C2	1	2	117.71	15.26	896	896	132.55	11.42	23505	23505	0.00
	C3	1	3	156.47	14.91	930	930	176.16	37.21	23856	23856	0.00
	C4	1	4	146.70	14.67	965	965	159.68	52.88	23294	23294	0.00
	C5	2	1	104.80	14.32	1344	1344	116.07	22.56	25701	25701	0.00
	C6	2	2	81.40	15.37	1382	1382	99.59	29.41	23804	23804	0.00
	C7	2	3	105.40	15.62	1417	1417	122.04	35.72	22088	22088	0.00
	C8	2	4	104.48	15.71	1453	1453	127.34	44.86	25050	25050	0.00

	C9	2	5	118.52	14.97	1487	1487	149.51	52.13	21305	21305	0.00
	C10	3	1	108.34	14.23	1836	1836	118.31	39.17	25052	25052	0.00
	C11	3	2	112.54	14.41	1870	1870	130.73	48.19	21968	21968	0.00
	C12	3	3	173.15	14.84	1907	1907	194.53	60.32	22776	22776	0.00
	C13	3	4	More than one hour	14.72	-	1938	More than an hour	71.43	-	21137	-
	C14	3	5	143.49	14.51	1979	1979	173.89	79.24	20420	20420	0.00
	C15	4	1	119.37	14.62	2335	2335	129.89	52.69	22845	22845	0.00
	C16	4	2	120.94	14.83	2373	2373	128.14	63.11	24503	24503	0.00
	C17	4	3	168.47	14.53	2408	2408	188.45	75.24	22535	22535	0.00
	C18	4	4	118.60	14.27	2444	2444	135.20	88.14	21392	21392	0.00
	C19	5	1	416.49	14.42	2893	2893	424.80	73.39	24187	24187	0.00
**(40, 4, 3, 5)**	C20	5	2	340.53	14.26	2928	2928	357.56	87.78	23706	23706	0.00
	C21	5	3	429.80	14.86	2960	2960	441.58	96.68	21551	21551	0.00
	C22	5	4	409.93	14.64	3005	3005	430.85	112.37	20858	20858	0.00
	C23	5	5	383.85	14.39	3055	3055	410.76	129.17	20101	20101	0.00
	C24	5	6	478.10	14.92	3106	3106	511.31	138.41	17850	17850	0.00
	C25	6	1	84.11	14.37	3342	3342	96.22	92.53	31912	31912	0.00
	C26	6	2	115.83	14.65	3379	3379	126.11	114.67	23830	23830	0.00
	C27	6	3	83.48	14.87	3425	3425	107.29	121.45	20761	20761	0.00
	C28	6	4	87.34	14.39	3468	3468	98.78	139.93	23938	23938	0.00
	C29	6	5	70.15	14.56	3515	3515	84.27	152.63	23795	23795	0.00
	C30	7	1	30.72	14.74	3815	3815	41.63	113.25	22849	22849	0.00
	C31	7	2	32.77	14.71	3852	3852	48.24	126.82	23796	23796	0.00
	C32	7	3	35.36	14.49	3893	3893	50.34	134.52	22502	22502	0.00
	C33	8	1	20.38	14.36	4343	4343	29.49	124.16	22465	22465	0.00
	C34	8	2	16.94	14.51	4378	4378	35.72	139.84	21385	21385	0.00
	C35	8	3	17.36	14.52	4473	4473	39.43	148.73	23336	23336	0.00
	C36	9	1	36.34	14.16	4993	4993	47.30	142.95	21786	21786	0.00
	C37	9	2	32.96	14.44	5042	5042	45.67	155.28	20447	20447	0.00
	C38	9	3	10.86	14.73	5081	5081	31.75	162.61	21591	21591	0.00
	C39	10	1	0.64	14.77	5603	5603	6.36	157.89	22670	22670	0.00
	C40	10	2	0.44	14.69	5668	5668	12.27	162.17	22678	22678	0.00

To compare the efficiency of the B&B and GA-SQP algorithms and decide which performs better. A one-way ANOVA test was conducted to determine whether both proposed algorithms performed similarly in terms of finding a good result in both stages. The factors in this analysis are GA-SQP and the B&B algorithm, and the results are CPU times and objective value. In this comparison, a box plot is used to demonstrate the efficacy of the proposed method. Since the aim of the model

in both stages is minimization, the optimal responses are the lowest values of objective value and CPU time. Table 1 illustrates the minimum objective value and CPU time achieved by GA-SQP and the B&B algorithms for stages 1 and 2. As shown in Table 1, the optimal values of the objective function obtained using GA-SQP are approximately the same as the values of the objective function obtained using B&B in small and medium-sized samples (in sets A, B, and C). However, as the size of the issues grows, the exact methods cannot solve them in a reasonable amount of time, while the hybrid GA-SQP can. Another important observation is that, except for C13, all instances in sets A, B, and C are optimized in at most 478.1 seconds using B&B, while GA achieves nearly identical results in at most 15.71 seconds in the first stage, which is significantly less than the CPU times observed by B&B. In the second stage, GA could achieve feasible solutions with similar objective values to B&B in less time on the CPU. In addition, C13 demonstrates that the CPU time required to solve the B&B is more than one hour because the instance size is roughly large. It's important to note that the B&B method is unable to solve the optimization problem in a reasonable amount of time due to the complexity of the developed model. In order to achieve a near-optimal solution, the GA algorithm is used. The convergence time of the B&B and GA-SQP algorithms for both stages is compared using a one-way ANOVA test with a significance level of 0.05 to determine the efficiency of algorithms in terms of how quickly the algorithm obtained the optimum solution. Table 2 shows the one-way ANOVA test results for each stage. According to the p-value in this table, there is a very significant difference in CPU times between the B&B and GA-SQP algorithms for both stages. This is identified by the p-value = 0.000 < α = 0.05 in the first stage and the p-value = 0.027 < α = 0.05 in the second stage. Figure 1 depicts a box plot of CPU time for the B&B and GA algorithms, demonstrating that the GA algorithm's CPU time is faster than the B&B algorithm in both stages of the proposed model.

Table 2 | ANOVA test results (Iran, 2022)

	DF	SS	MS	F	P
**First stage**	1	198241	198241	25.41	0.000
	S = 88.32	R-Sq = 19.05%		R-Sq(adj) = 18.30%	
**Second stage**	1	46239	46239	5.04	0.027
	S = 95.83	R-Sq = 4.45%		R-Sq(adj) = 3.57%	

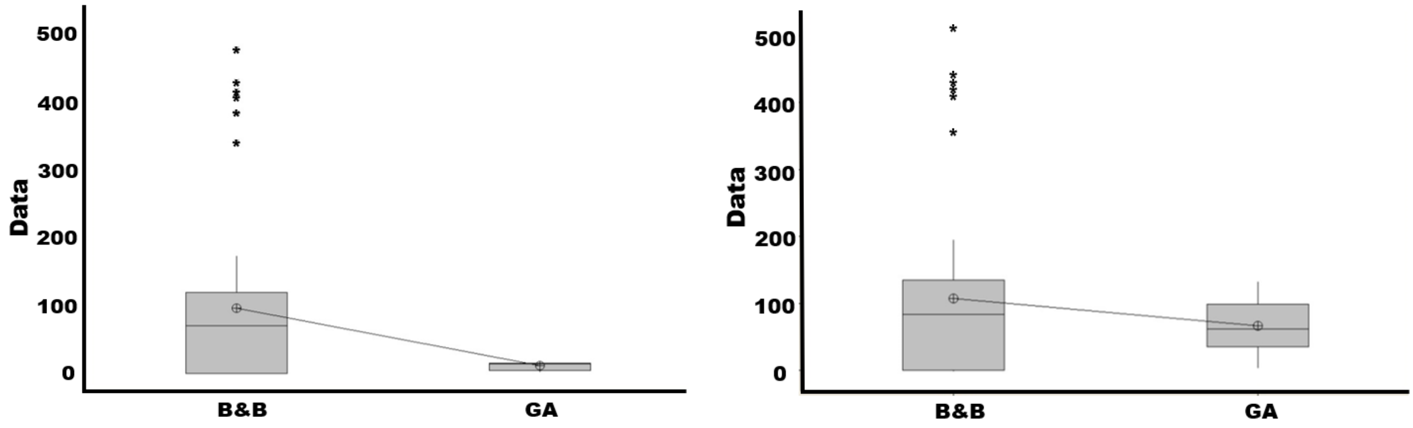

Stage 1 **Stage 2**

Figure 1 | The Box plot of CPU time for the Branch and bound and Genetic algorithms (Iran, 2022)

In this study, some small and medium-sized instances were used to evaluate the performance of B&B and GA-SQP in solving the issue of two-stage location-allocation in healthcare services. Comparing the values of the objective functions obtained by the B&B and GA-SQP algorithms shows the effectiveness of the algorithms for each instance. As shown in Table 1, the optimal values of the objective function obtained using GA-SQP are approximately the same as the values of the objective function obtained using B&B. We can conclude that the methods under consideration are indifferent. This means that GA and B&B perform similarly for both stages in terms of effectiveness. except for C13, where the B&B approach was unable to determine the

optimal solution due to the complexity of the developed model. It is important to note that, in large instances, the B&B method is unable to solve the optimization problem in a reasonable amount of time. As a result, the GA method is used to obtain a near-optimal solution. In comparison to B&B, GA-SQP is the fastest algorithm that converges to the lowest cost. One of the considerations for very large-scale location-allocation problems in healthcare is the efficiency of algorithm processing time.
