# Supplementary material for: A Two-Stage Optimization Approach for Healthcare Facility Location- Allocation Problems With Service Delivering Based on Genetic Algorithm
Source: Int J Public Health. 2023 Feb 28;68:1605015. doi: 10.3389/ijph.2023.1605015 (PMC10011119; doi:10.3389/ijph.2023.1605015)
Supplement: Supplementary file 4 [file DataSheet3.pdf]

Numerical Example Data

In this numerical example, ten population centers are considered, which are candidate locations for LLHCCs or HLHCCs, and the demand for either or both. Three types of services (Service 1, Service 2, and Service 3) are considered in this example and represented by n = 3. These services are available at two capacity levels (m = 2). In the current study, an extended planning horizon included three periods. The capacity levels for each of the three types of services are shown in Table 1.

Table 1 | m^{th} capacity level for the nth service (Iran, 2022)

Service type	Capacity level 1	Capacity level 2
**1**	18	30
**2**	20	35
**3**	12	25

Table 2 depicts the variable cost of transferring the nth type of service to the kth HLHCC at the mth capacity level. The minimum number of HLHCCs and LLHCCs that must be covered by all nodes for each type of service is 1 and 2, respectively. In addition, Table 3 also provides information on the fixed cost of establishing the HLHCC and the LLHCC.

Table 2 | The variable cost of transferring the nth type of service to the kth higher-level healthcare center at the mth capacity level (Iran, 2022)

HLHCC	Capacity level 1			Capacity level 2		
	Service type			Service type		
	**1**	**2**	**3**	**1**	**2**	**3**
**1**	19	12	17	10	18	15
**2**	19	17	13	14	20	17
**3**	18	16	17	12	10	17
**4**	11	11	10	19	15	18
**5**	16	17	20	14	14	17
**6**	12	13	16	19	15	10
**7**	18	12	12	12	18	19
**8**	20	19	10	14	15	14
**9**	10	14	15	19	10	14
**10**	19	11	20	15	18	19

Table 3 | The fixed cost of establishing the higher-level healthcare centers and the lower-level healthcare centers (Iran, 2022)

**Node *i***	F_k	f_j
**1**	197	10
**2**	186	12
**3**	105	17
**4**	181	13
**5**	127	14
**6**	101	14
**7**	135	12
**8**	163	14
**9**	136	18
**10**	151	13

The variable costs of transferring the first, second, and third types of services from HLHCCs and LLHCCs to the demand point are shown in Tables 4 and 5. Furthermore, the variable cost of transferring the first, second, and third types of services from an HLHCC to an LLHCC within its coverage radius is shown in Table 6. The coverage radius of healthcare centers is set to zero or one, depending on whether demand nodes are located within these coverage radiuses for receiving services. For receiving the first, second, and third types of services, the coverage radius of HLHCCs and LLHCCs is uniformly distributed between zero and one. Other problem parameters, such as the shortage cost of type n service for demand points, demand nodes of n-type service in the first, second, and third time periods, and transportation costs among LLHCCs, are shown in Tables 7, 8, and 9.

Table 4 | The variable cost of transferring the first, second, and third types of services from higher-level healthcare centers to the demand point (Iran, 2022)

Demand point	First type of services										Second type of services										Third type of services									
	**1**	**2**	**3**	**4**	**5**	**6**	**7**	**8**	**9**	**10**	**1**	**2**	**3**	**4**	**5**	**6**	**7**	**8**	**9**	**10**	**1**	**2**	**3**	**4**	**5**	**6**	**7**	**8**	**9**	**10**
1	8	4	8	12	4	8	16	4	12	12	12	4	8	12	8	8	8	12	16	16	4	16	16	4	8	8	16	8	12	8
2	12	8	16	4	8	16	4	16	4	8	16	16	4	12	8	8	4	12	8	8	8	8	8	12	8	12	12	16	4	12
3	4	8	12	8	4	12	12	12	12	12	4	16	12	8	4	16	12	16	16	4	12	16	4	12	8	4	4	4	16	12
4	16	8	16	12	8	8	4	16	12	8	4	8	16	8	8	4	12	16	12	12	12	4	4	4	12	4	12	8	16	16
5	16	16	12	4	12	12	4	16	4	4	4	12	16	16	16	16	4	8	16	4	4	16	12	8	4	8	8	8	16	16
6	8	12	4	12	16	16	4	4	16	12	4	8	8	16	12	4	8	4	16	12	4	8	4	4	4	8	4	8	4	16
7	12	16	4	8	16	8	4	8	16	16	8	16	16	16	4	4	16	12	16	12	12	8	8	8	12	4	4	12	8	16
8	16	4	12	8	4	12	16	12	4	8	12	4	16	16	12	4	8	16	8	8	4	4	4	12	12	4	16	8	8	4
9	12	8	12	16	8	4	12	16	12	8	16	4	12	12	12	12	4	12	16	16	16	8	4	4	8	8	8	4	16	8
10	12	12	8	4	4	16	12	16	12	8	4	12	8	8	8	8	4	12	8	12	4	16	16	12	16	4	12	12	8	8

Table 5 | The variable cost of transferring the first, second, and third types of services from lower-level healthcare centers to the demand point (Iran, 2022)

Demand point	First type of services										Second type of services										Third type of services									
	**1**	**2**	**3**	**4**	**5**	**6**	**7**	**8**	**9**	**10**	**1**	**2**	**3**	**4**	**5**	**6**	**7**	**8**	**9**	**10**	**1**	**2**	**3**	**4**	**5**	**6**	**7**	**8**	**9**	**10**
**1**	3	1	3	1	2	4	2	4	1	3	3	4	4	2	3	3	3	2	2	1	3	4	1	1	2	1	3	3	1	3
**2**	4	2	3	2	2	3	1	4	1	3	3	4	1	1	1	4	4	2	3	3	4	2	4	4	2	4	1	2	2	4
**3**	4	2	2	3	4	3	4	1	1	1	2	4	1	3	1	3	1	3	1	3	1	1	3	1	1	2	3	1	1	3
**4**	1	2	1	3	1	2	2	1	2	1	4	3	4	4	1	3	2	4	2	4	2	2	3	2	1	2	1	4	2	1
**5**	3	2	3	2	4	3	2	4	2	4	4	2	4	2	3	2	2	3	3	4	2	4	1	2	4	4	4	1	4	2
**6**	3	2	2	1	2	3	4	3	3	3	4	1	4	2	4	2	4	2	4	4	1	2	4	4	1	3	3	1	3	1
**7**	4	3	2	1	3	1	4	1	2	2	3	2	1	3	4	4	4	2	4	1	3	3	3	2	4	4	1	4	3	4
**8**	1	1	3	1	2	3	1	3	3	2	2	4	1	1	4	4	4	1	3	2	1	2	4	3	2	1	3	2	1	4
**9**	1	2	1	1	4	3	4	1	2	1	4	2	2	1	3	3	3	1	4	2	3	3	1	4	4	4	1	1	4	2
**10**	2	4	3	2	1	3	3	3	2	3	2	1	4	4	4	4	1	3	4	1	4	2	1	1	2	3	2	3	3	3

Table 6 | The variable cost of transferring the first, second, and third types of services from a higher-level healthcare center to a lower-level healthcare center is covered (Iran, 2022)

HLHCC	first type of services										second type of services										third type of services									
	**1**	**2**	**3**	**4**	**5**	**6**	**7**	**8**	**9**	**10**	**1**	**2**	**3**	**4**	**5**	**6**	**7**	**8**	**9**	**10**	**1**	**2**	**3**	**4**	**5**	**6**	**7**	**8**	**9**	**10**
**1**	3	4	3	2	1	4	1	4	3	4	2	1	1	2	1	2	4	4	3	4	2	4	3	2	1	3	1	1	3	1
**2**	2	4	4	4	1	2	2	1	4	2	3	3	4	4	1	3	1	3	2	3	3	4	2	2	1	4	2	3	1	1
**3**	1	4	1	1	2	4	3	2	4	1	3	1	4	1	3	4	2	4	1	3	1	3	3	4	2	4	2	1	4	2
**4**	1	3	4	1	4	1	1	1	1	4	1	3	1	1	1	3	3	3	3	3	1	3	4	4	4	2	4	4	3	2
**5**	1	3	4	2	3	1	4	1	3	1	2	4	1	1	1	2	1	3	1	3	4	2	2	2	1	1	2	2	3	4
**6**	3	3	2	4	3	3	4	1	3	3	4	3	1	4	4	4	4	4	1	3	1	1	4	2	4	4	4	2	1	3
**7**	4	2	3	4	4	3	2	3	3	1	4	2	2	3	4	2	3	1	2	2	2	4	1	2	4	1	4	4	2	2
**8**	3	4	4	3	4	3	1	4	3	3	2	1	1	4	3	1	4	4	1	3	1	4	4	2	3	3	4	1	2	4
**9**	1	1	2	4	3	1	2	3	4	4	2	4	3	1	4	3	4	1	4	4	4	3	4	4	1	2	2	1	2	2
**10**	1	3	2	1	4	2	4	1	2	3	3	1	3	4	3	1	4	2	3	3	2	1	3	4	2	2	4	1	1	3

Table 7 | Shortage cost of type *n* service for demand point *i* (Iran, 2022)

Node *i*	Service type		
	**1**	**2**	**3**
**1**	14	13	15
**2**	15	13	14
**3**	15	12	14
**4**	13	13	13
**5**	12	14	11
**6**	13	15	13
**7**	14	14	11
**8**	14	15	13
**9**	15	12	15
**10**	14	14	11

Table 8 | The demand of node *i* of *n*-type service in the first, second, and third time period (Iran, 2022)

Node *i*	Time period 1			Time period 2			Time period 3		
	Service type			Service type			Service type		
	1	2	3	1	2	3	1	2	3
**1**	14	13	15	14	13	15	14	13	15
**2**	15	13	14	15	13	14	15	13	14
**3**	15	12	14	15	12	14	15	12	14
**4**	13	13	13	13	13	13	13	13	13
**5**	12	14	11	12	14	11	12	14	11
**6**	13	15	13	13	15	13	13	15	13
**7**	14	14	11	14	14	11	14	14	11
**8**	14	15	13	14	15	13	14	15	13
**9**	15	12	15	15	12	15	15	12	15
**10**	14	14	11	14	14	11	14	14	11

Table 9 | Transportation costs among the lower level healthcare centers (Iran, 2022)

LLHCC	LLHCC									
	1	2	3	4	5	6	7	8	9	10
**1**	3	3	1	3	4	2	4	4	3	1
**2**	3	1	2	3	3	4	2	4	3	3
**3**	1	2	1	3	1	4	2	2	2	3
**4**	1	1	2	4	1	4	2	4	1	1
**5**	4	3	1	3	3	1	2	4	1	2
**6**	3	4	1	4	2	2	3	3	2	4
**7**	1	4	1	2	3	1	4	1	4	2
**8**	4	3	2	2	1	4	3	1	2	1
**9**	3	3	4	3	1	2	1	1	4	1
**10**	4	2	1	2	1	1	4	1	2	2

Experimental Results

The presented model has been coded in Lingo 17 in this numerical example. The coded model was solved by the Core i7 processor. Although the facility location has been known as an NP-hard problem, the software could solve the model by the exact method and obtain the global optimum according to the type of modeling. In the first stage, the model aims to effectively identify the

locations of LLHCCs and HLHCCs as well as the capacities of each service provided at these locations. Allocations and inter-service referrals are executed for each period in the second stage. According to the results of the first stage, an HLHCC was established in node 3, and three LLHCCs were established in nodes 1, 2, and 9. In addition, the HLHCC offers services 1 and 2 at capacity level 2, and service 3 at capacity level 1.

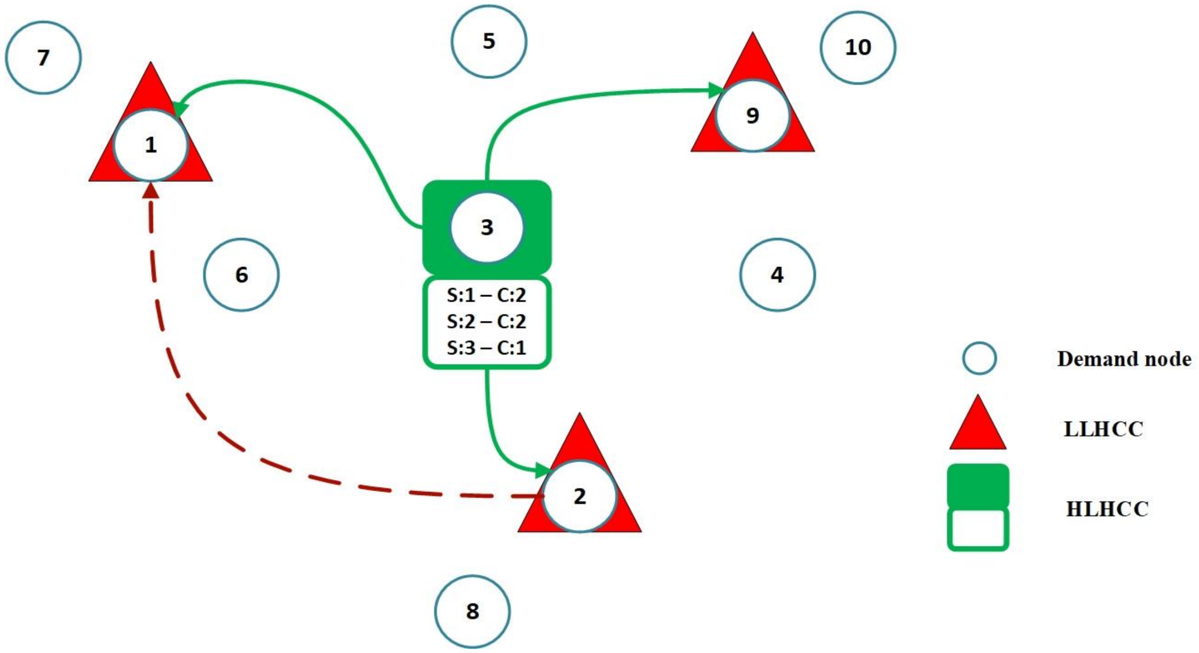

Figure 1 | The schematic representation of the numerical example's outcome (Iran, 2022)

The services are transferred to LLHCCs via HLHCCs in the second stage of the proposed model. As shown in Figure 1, LLHCCs 1, 2, and 9 receive services from HLHCC 3, denoted by the green line. The transfer of services occurs from LLHCCs 1 to 2, which is similar in all three time periods in the proposed numerical example, as shown by the red dashed line in Figure 1. Table 10 shows the rate of transfer of type *n* service from the mentioned HLHCC to LLHCCs with a 3D element in three time periods.

Table 10 | Number of services transferred from higher-level healthcare centers to lower-level healthcare centers over a given time period (Iran, 2022)

LLHCC	Time period		
	1	2	3
1	[22,0,7]	[24,0,10]	[21,0,4]
2	[0,0,0]	[0,0,0]	[0,0,0]
9	[4,0,0]	[3,0,0]	[4,0,0]

Table 11 shows the rate of type-*n* service transfer from the kth HLHCC to the jth demand point and the rate of type-*n* service transfer from the jth LLHCC to the ith demand point over three time periods. As seen in the table, the rows represent demand nodes, and the columns represent the locations of LLHCCs and HLHCCs chosen from the demand points over time periods. The HLHCC is located at node 3, and the LLHCCs are located at nodes 1, 2, and 9.

Table 11 | Transfer amounts of the first, second, and third types of services to the demand nodes (Iran, 2022)

Demand point	Location of LLHCCs and HLHCCs											
	1			2			3			9		
	Time period			Time period			Time period			Time period		
	1	2	3	1	2	3	1	2	3	1	2	3
1	-	-	-	-	[0,0,1]	-	[5,4,0]	[3,3,0]	[4,5,0]	-	-	-
2	[3,0,0]	[3,0,0]	[3,0,0]	[0,0,3]	[0,0,5]	[0,0,3]	-	-	-	-	-	-
3	[1,0,0]	[4,0,0]	[4,0,0]	-	-	-	[0,3,5]	[0,5,2]	[0,0,5]	-	-	-
4	[1,0,0]	[4,0,0]	[4,0,0]	-	-	-	-	-	-	-	-	-
5	[4,0,0]	[2,0,0]	-	-	-	-	[0,3,0]	[0,3,0]	[0,3,0]	-	-	-
6	[3,0,0]	-	-	-	-	-	-	-	-	-	-	-
7	[5,0,0]	[2,0,0]	[5,0,0]	-	-	-	[0,1,0]	[0,4,0]	[0,4,0]	-	-	-
8	-	-	-	-	-	-	[0,0,3]	-	-	[4,0,0]	[3,0,0]	[4,0,0]
9	[1,0,0]	[4,0,0]	[2,0,0]	[0,0,1]	[0,0,4]	[0,0,4]	-	-	-	-	-	-
10	[3,0,0]	[5,0,0]	[4,0,0]	-	-	-	[0,5,0]	[0,2,0]	[0,5,0]	-	-	-

Each cell in the table shows a 3D element. The first element represents the first type of service; the second element shows the second type of service; and the third one presents the third type of service. For example, the cell in the table's second row and first column indicates that the second

node of demand receives the first type of service in the amount of 3 units from LLHCC, which is located in the first node, during the first time period. The shortage of the nth type of service for each demand node and the difference between the maximum and minimum value of the nth type of service shortage in all nodes over three time periods are shown in Tables 12 and 13, respectively.

Table 12 | The node *i* shortage of the nth type of service in time period (Iran, 2022)

	**Time period 1**			**Time period 2**			**Time period 3**		
**Node *i***	**Service type**			**Service type**			**Service type**		
	**1**	**2**	**3**	**1**	**2**	**3**	**1**	**2**	**3**
**1**	0	0	5	0	0	2	0	0	4
**2**	0	2	0	0	4	0	0	1	0
**3**	0	4	0	0	0	0	0	0	0
**4**	0	0	5	0	0	3	0	0	4
**5**	5	0	2	0	0	4	0	0	1
**6**	3	4	4	5	4	4	0	1	0
**7**	0	0	4	0	0	3	0	0	5
**8**	1	5	3	0	2	3	0	4	3
**9**	0	3	0	0	5	0	0	3	0
**10**	0	0	4	0	0	4	0	0	1

Table 13 | The difference between the maximum and the minimum value of the nth service shortage in all nodes (Iran, 2022)

**Service type**	**Time period**		
	**1**	**2**	**3**
**1**	1	1	0
**2**	1	1	1
**3**	1	1	1
