# Supplementary material for: A Two-Stage Optimization Approach for Healthcare Facility Location- Allocation Problems With Service Delivering Based on Genetic Algorithm
Source: Int J Public Health. 2023 Feb 28;68:1605015. doi: 10.3389/ijph.2023.1605015 (PMC10011119; doi:10.3389/ijph.2023.1605015)
Supplement: Supplementary file 5 [file DataSheet1.pdf]

Assumptions

1. LLHCCs and HLHCCs are selected among demand points.
2. Only one health center can be in each node.
3. The capacity level of HLHCCs is specified for each type of service.
4. Each LLHCC is assigned to only one HLHCC, and appropriate transportation facilities should also be provided for all HLHCCs.
5. The cost of transportation among points of demand has been calculated based on the value of the flow and the distance between the points, and it has been assumed that the cost of transportation is fixed and remains constant.
6. HLHCCs and LLHCCs have a specified covering radius.
7. There is no direct connection between two nodes that are not within the same radius of coverage.
8. The model specifies the location and number of facilities. If a facility has already been built, it can be added to the model.
9. The shortage is allowed.
10. The presented model is a multi-period model.
11. The demand is definite.

Sets

I	The set of demand nodes ($i \in I$)
J	The set of LLHCCs ($j, j' \in J, J \subseteq I$)
K	The set of HLHCCs ($K \subseteq I$)
T	The set of periods ($t \in T$)
N	The set of services ($n \in N$)

Parameters

r_{2k}	Covering radius of HLHCC $k \in I$
r_{1j}	Covering radius of LLHCC $j \in I$
m	Capacity level
M	A large positive number
$C_{jj'}$	Transportation costs among LLHCCs j and j'
$\hat{c}_{nm}$	The mth capacity level for the nth service
C''_{kjn}	The variable cost of transferring the nth type of service from the kth HLHCC to the jth covered LLHCC
q'_{ikn}	The variable cost of providing the nth type of service from the kth HLHCC to the ith demand node
q_{ijn}	The variable cost of providing the nth type of service from the jth LLHCC to the ith demand node
$C'_{jj'n}$	The variable cost of transferring the nth type of service from the jth LLHCC to the j'^{th} LLHCC
G_{knm}	The variable cost of providing the nth type of service to the kth HLHCC at the mth capacity level
b_{ikn}	1 if the ith demand node for receiving the nth service is within the kth HLHCC covering radius, 0 otherwise
a_{ijn}	1 if the ith demand node for receiving the nth service regarding the covering radius (r_{1j}) was under the coverage of jth LLHCC, 0 otherwise
D_{int}	The demand for node i from n^{th} type service in period t
F_k	The fixed cost of establishing an HLHCC at demand node k
f_j	The fixed cost of establishing an LLHCC at node j
A_n	1 if the n type service is a HLHCC service, 0 otherwise
P_{in}	The cost of n type service shortage for demand node i
d_{ik}	The distance among node i and node k
L_n	The cost of the difference among the highest and the lowest value of the nth service shortage in all nodes
α_n^{max}	The maximum difference among the maximum and the minimum value of the nth service shortage in all nodes

V The minimum number of LLHCCs that must be covered by all nodes for each type of service

H The minimum number of HLHCCs that must be covered by all nodes for each type of service

Decision Variables

y_j 1 if an LLHCC is established at node *j*, 0 otherwise

X_{jk} 1 if LLHCC *j* is assigned to HLHCC *k*, 0 otherwise

X_{kk} 1 if the *k* HLHCC is set up in *k* node, 0 otherwise

Z_{knm} 1 if the kth HLHCC provides the nth service at the mth capacity, 0 otherwise

W_{kjnt} The amount of the nth type of service transferred from the kth HLHCC to the jth LLHCC in period *t*

$h_{jj'nt}$ The amount of the nth type of service transferred from j^{th} LLHCC to j'^{th} LLHCC in period *t*

e_{jint} The amount of nth type of service provided from the jth LLHCC to the ith demand point in period *t*

B_{int} The node *i* shortage of the nth type of service in period *t*

u_{kint} The amount of the nth type of service provided from the kth HLHCC to the ith demand point in period *t*

$x_{jj't}$ 1 if the LLHCC *j* is connected to the LLHCC *j'* in period *t*, 0 otherwise

δ_{nt} The minimum amount of the nth service shortage in all nodes in period *t*

β_{nt} The maximum amount of the nth service shortage in all nodes in period *t*

α_{nt} The difference among the maximum and the minimum value of the nth service shortage in all nodes in period *t*
