# Supplementary material for: A Two-Stage Optimization Approach for Healthcare Facility Location- Allocation Problems With Service Delivering Based on Genetic Algorithm
Source: Int J Public Health. 2023 Feb 28;68:1605015. doi: 10.3389/ijph.2023.1605015 (PMC10011119; doi:10.3389/ijph.2023.1605015)
Supplement: Supplementary file 6 [file DataSheet5.pdf]

Parameters Tuning

The values of any meta-heuristic algorithm's parameters determine its effectiveness and quality. Different parameter combinations in an algorithm can produce solutions of varying quality. In this study, the Taguchi approach is used to tune the algorithmic parameters. This method significantly reduces the number of tuning experiments required. A statistical measure known as the signal-to-noise ratio (S/N) is calculated in this method to evaluate performance. To ensure that the experiments are carried out fairly, the number of function evaluations is fixed for each experiment in this study. The function evaluation number is calculated based on the number of iterations $\times$ population size in the GA algorithm. As a result, the Taguchi methods are used to tune the parameters population size, crossover rate, and mutation rate, and the number of iterations equal to (function evaluation number / population size) is considered. The parameter levels of the GA algorithm for stage 1 are reported in Table 1. In this study, the L9 design of the Taguchi method is employed. The obtained response values of the GA algorithm for stage 1 in each experiment are shown in Table 2. The related S/N ratio, which is calculated by the Minitab 17 software, is presented in Figure 1. In this figure, the best level of each parameter is chosen to be the one with the highest S/N ratio. As a result, the proper values of the algorithm parameters are reported in Table 3.

Table 1 | Parameter levels of the Genetic algorithm for stage 1 (Iran, 2022)

Parameter	Parameter range	Level 1	Level 2	Level 3
Population size	100-300	100	200	300
Crossover rate	0.7-0.9	0.7	0.8	0.9
Mutation rate	0.1-0.3	0.1	0.2	0.3

Table 2 | Obtained response values of the Genetic algorithm for stage 1 (Iran, 2022)

Run order	Population size	Crossover rate	Mutation rate	Objective Value
1	100	0.7	0.1	851
2	100	0,8	0.2	875
3	100	0.9	0.3	853
4	200	0.7	0.2	860
5	200	0,8	0.3	892
6	200	0.9	0.1	855
7	300	0.7	0.3	861
8	300	0,8	0.1	870
9	300	0.9	0.2	867

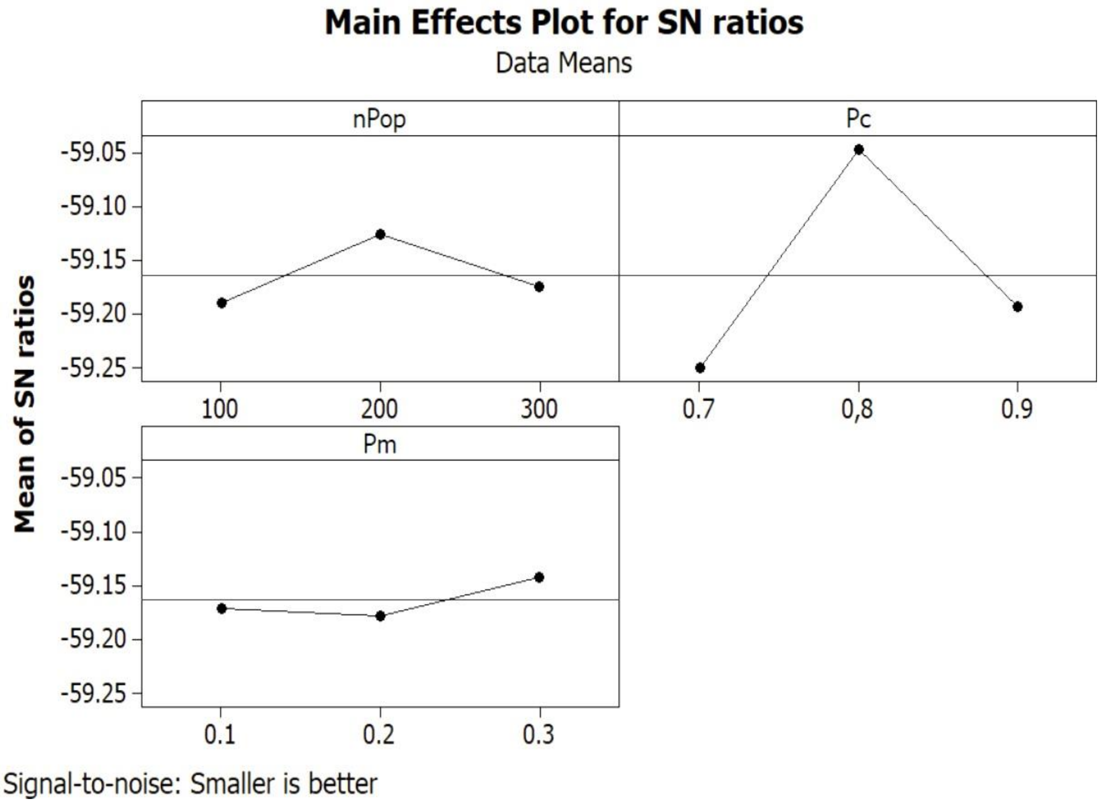

Figure 1 | Signal-to-noise ratios of the Genetic algorithm for stage 1 (Iran, 2022)

Table 3 | Proper values of parameters of the Genetic algorithm for stage 1 (Iran, 2022)

Parameter	Value
Population size	200
Crossover rate	0.8
Mutation rate	0.3

The parameter levels and the obtained response values of the GA algorithm for stage 2 are shown in Tables 4 and 5, respectively. Figure 2 depicts the relevant S/N ratio as estimated by the Minitab 17 software. The best level of each parameter is chosen in this figure to have the maximum S/N ratio. The appropriate algorithm parameter values are presented in Table 6.

Table 4 | Parameter levels of the Genetic algorithms for stage 2 (Iran, 2022)

Parameter	Parameter range	Level 1	Level 2	Level 3
Population size	100-300	100	200	300
Crossover rate	0.7-0.9	0.7	0.8	0.9
Mutation rate	0.1-0.3	0.1	0.2	0.3

Table 5 | Obtained response values of the Genetic algorithm for stage 2 (Iran, 2022)

Run order	Population size	Crossover rate	Mutation rate	Objective Value
100	0.7	0.1	1582	100
100	0.8	0.2	1570	100
100	0.9	0.3	1585	100
200	0.7	0.2	1591	200
200	0.8	0.3	1595	200
200	0.9	0.1	1598	200
300	0.7	0.3	1592	300
300	0.8	0.1	1596	300
300	0.9	0.2	1581	300

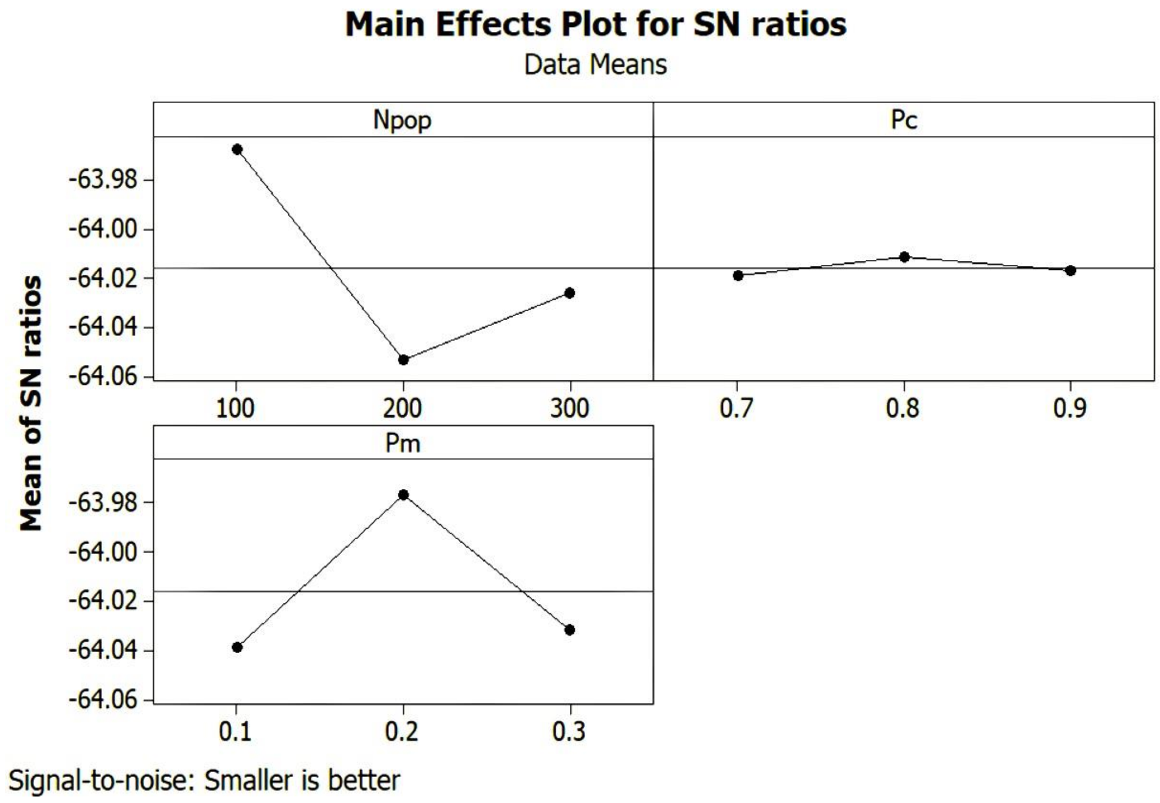

Figure 2 | Signal-to-noise ratios of the Genetic algorithm for stage 2 (Iran, 2022)

Table 6 | Proper values of parameters of the Genetic algorithm for stage 2 (Iran, 2022)

Parameter	Value
Population size	100
Crossover rate	0.8
Mutation rate	0.2
